# CRISPR/Cas13a: Compensatory Target Activation Mechanism

**DOI:** 10.1002/advs.202524156

**Published:** 2026-04-09

**Authors:** Bowen Jiang, Tenghua Zhang, Yao Lu, Shujun Zhou, Wenjing Xiao, Shuming Pan, Nan Shi, Yan Sheng, Jiaming Hu

**Affiliations:** ^1^ International Joint Laboratory of Catalytic Chemistry, Innovation Institute of Carbon Neutrality, Department of Chemistry, College of Sciences Shanghai University Shanghai China; ^2^ MOE Key Laboratory of Laser Life Science & Institute of Laser Life Science, Guangdong Provincial Key Laboratory of Laser Life Science, College of Biophotonics South China Normal University Guangzhou China; ^3^ Emergency Department, Putuo Hospital Shanghai University of Traditional Chinese Medicine Shanghai China; ^4^ Institute of Translational Medicine Shanghai University Shanghai China

**Keywords:** compensatory target activation, CRISPR/Cas13a, CRISPR/Cas13a‐CTAM, double‐effector, immobilized Cas13a

## Abstract

CRISPR/Cas13a is a powerful RNA‐targeting platform for molecular diagnostics, but conventional single‐effector systems typically require contiguous RNA targets longer than ∼20–28 nt, limiting sensitivity and target flexibility. CRISPR/Cas13a‐CTAM is presented as a compensatory target activation mechanism that facilitates synergistic Cas13a activation through two independently programmable short RNA effectors. By functionally decoupling allosteric activation and binding stabilization, CRISPR/Cas13a‐CTAM supports robust activation by ultra‐short RNA targets as short as 13 nt, substantially expanding the detectable target range. Compared with traditional single‐effector Cas13a assays, CRISPR/Cas13a‐CTAM achieves a detection limit of 1 fM for a 13‐nt RNA target, representing an approximately tenfold sensitivity improvement. Notably, a single‐nucleotide mismatch within the 13‐nt target induces up to a 35‐fold reduction in apparent cleavage rate, corresponding to a sevenfold enhancement in mismatch discrimination. The dual‐effector architecture further enables simultaneous dual‐target detection, demonstrated by dual miRNA profiling related to COVID‐19 and combined detection of exosome membrane proteins. Moreover, the weakly activating effector was utilized as an anchoring module to achieve the first functional immobilization of Cas13a on a sensing surface, enabling in situ electrochemical miRNA detection. By overcoming the reliance on long RNA targets, CRISPR/Cas13a‐CTAM provides a sensitive, programmable platform for RNA diagnostics and integrated biosensor development.

## Graphical abstract

CRISPR/Cas13a‐CTAM as a novel compensatory target activation mechanism that enables synergistic activation of Cas13a via two independently editable short RNA effectors. This dual‐effector system maintains enzymatic activity comparable to traditional single‐effector while significantly enhancing flexibility, sensitivity, and application scope. Key advancements include the ability to detect single‐base mismatches, ultra‐short RNAs, and conduct multi‐target detection, alongside the first successful immobilization of Cas13a on sensing surfaces.

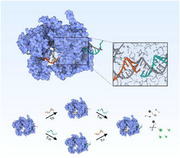

## 1 Introduction

The CRISPR/Cas system, consisting of clustered, regularly interspaced short palindromic repeats (CRISPR) and CRISPR‐associated proteins (Cas) [1], serves as an adaptive immune mechanism in prokaryotes, protecting against invading genetic elements such as viruses and plasmids. Guided by CRISPR RNA (crRNA), the system precisely recognizes, binds to, and cleaves foreign nucleic acids, allowing for targeted disruption of exogenous DNA or RNA sequences [2, 3, 4, 5]. Among the various CRISPR/Cas systems, Cas13a, a single‐component effector from the type VI class 2 CRISPR system, is distinct for its RNA‐targeting capability [6, 7]. Unlike DNA‐targeting CRISPR systems, Cas13a specifically targets RNA, making it valuable for RNA‐related diagnostics and therapeutics. Upon binding to target RNA via complementary crRNA, Cas13a activates a trans‐cleavage mechanism that indiscriminately cleaves nearby non‐target RNA molecules [7, 8, 9, 10, 11]. This trans‐cleavage activity has been utilized for various applications, especially in developing sensitive molecular diagnostics by amplifying detection signals. Recently, structural studies have offered important insights into Cas13a activation. High‐resolution techniques such as cryo‐electron microscopy have revealed conformational changes associated with target binding and catalytic domain activation, clarifying the structural basis of Cas13a's allosteric regulation [12, 13]. These advances have facilitated the development of Cas13a‐based biosensing and precision diagnostic tools [14, 15, 16].

Nevertheless, most current Cas13a assays still rely on a conventional activation scheme in which a single, contiguous RNA effector—typically longer than ∼28 nucleotides—forms an extended duplex with the target to trigger trans‐cleavage. Although effective, this design can restrict the range of detectable targets and the flexibility of assay formats. First, efficiently detecting short RNA targets such as miRNAs (∼22 nt) remains challenging, as the limited base‐pairing length may not be sufficient to induce strong Cas13a activation, especially under stringent conditions. Second, the indiscriminate trans‐cleavage activity of Cas13a, triggered by a single effector RNA, significantly limits its ability to specifically detect multiple RNA targets in a single reaction mixture. This non‐specific cleavage often results in background noise, necessitating additional steps or separate reactions for multiplex detection. Third, integrating Cas13a into solid‐state biosensors continues to be difficult. Inappropriate immobilization methods may compromise enzymatic activity or elevate background noise, and the sensing interface struggles to sustain controllable activation of the enzyme. In other words, a technical barrier persists between stable enzyme attachment and controllable signal transduction. These constraints highlight the need for more flexible Cas13a activation mechanisms that extend beyond the single contiguous effector–target duplex paradigm.

Notably, recent efforts such as our team's CRISPR‐Cas13a Gemini system have demonstrated noncanonical activation architectures that enable dual‐input RNA sensing through the assembly of two Cas13a:crRNA complexes in a fist‐to‐fist configuration. However, such strategies rely on dual effector enzymes and higher‐order complex formation, which may increase system complexity and limit modular programmability.

**FIGURE 1:**
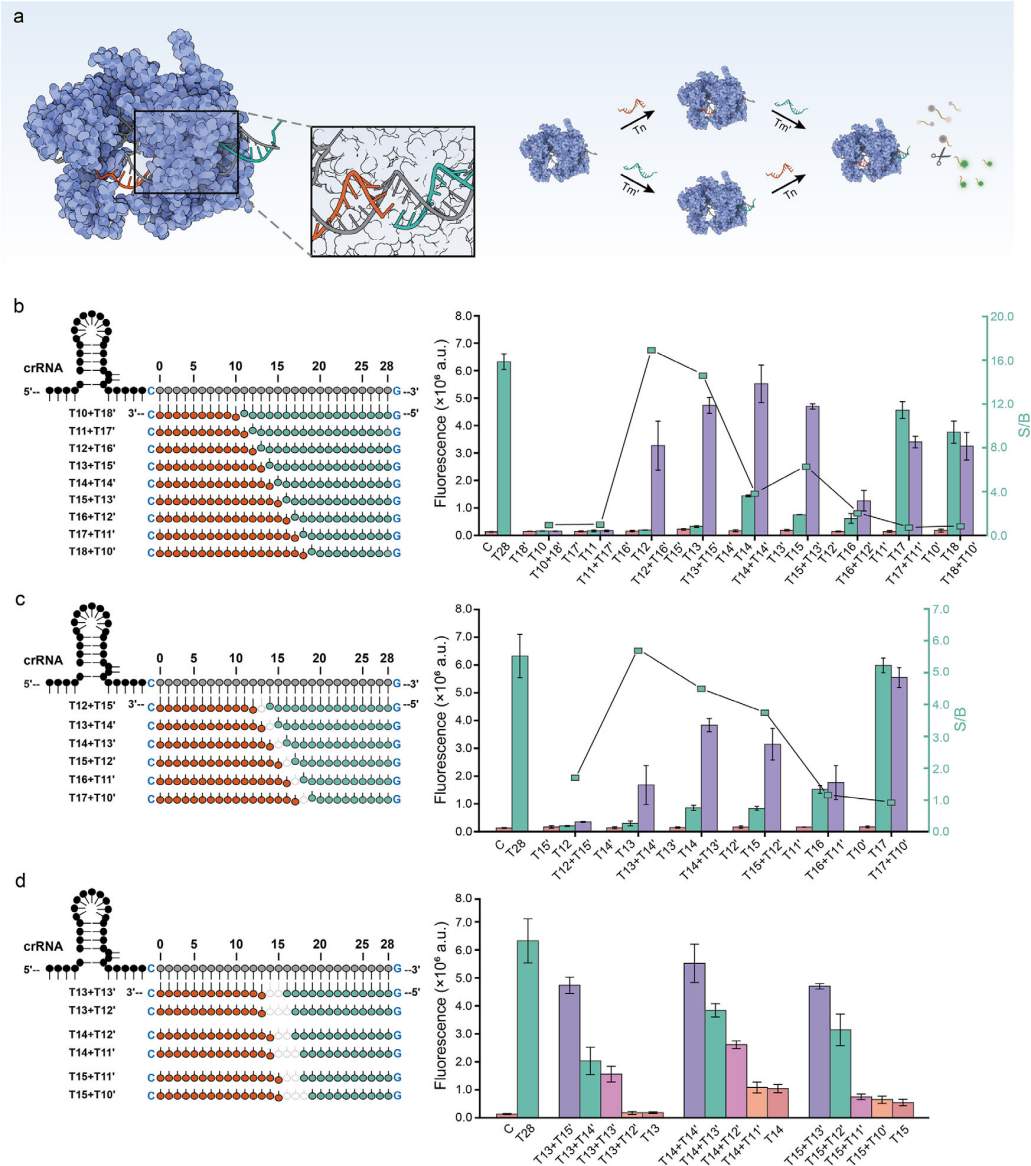
The synergistic relationship between Tn and Tm'. (a) Schematic diagram of CRISPR/Cas13a‐CTAM, which employs a compensatory target activation mechanism, enabling the synergistic activation of Cas13a by two independently editable double‐effector. (b) Schematic representation of the double‐effector combination series and their respective effects on CRISPR/Cas13a activation. S/B refers to the ratio of Tn + Tm' to Tn. (c) Schematic representation of the double‐effector combinations with single‐base deletions and their respective effects on CRISPR/Cas13a activation. (d) Double‐effector combinations with multi‐base continuous deletions. Representative time‐course data can be found in Figures S2–S4. The data are expressed as mean ± SD, n = 3, with three independent replicates.

Here, we report a different complementary activation route: Cas13a can be synergistically activated by two independent, short RNA effectors within a single effector complex (Figure 1a). We term this mechanism CRISPR/Cas13a Compensatory Target Activation Mode (CRISPR/Cas13a‐CTAM). Importantly, CRISPR/Cas13a‐CTAM was not initially conceived as a design solution to any single limitation. Rather, it emerged during systematic truncation and variant testing, where we found that two short effectors (each individually insufficient or weak in triggering activation) can cooperatively activate Cas13a only when co‐engaged. Building on this observation, we formalize CRISPR/Cas13a‐CTAM as a distinct compensatory activation mechanism that differs fundamentally from the traditional contiguous single‐effector model and Gemini system. By distributing the activation requirement between two programmable short effectors, CRISPR/Cas13a‐CTAM introduces new design flexibility that overcomes the key limitations of conventional Cas13a: Sensitive detection of short RNAs with improved single‑nucleotide mismatch discrimination, simultaneous dual miRNA profiling related to COVID‑19, combined detection of two protein biomarkers on exosome surfaces, and electrochemical miRNA sensing via Cas13a immobilized on an electrode by one weak activation effector as an anchor.

In summary, CRISPR/Cas13a‐CTAM provides a practical, programmable dual‐effector activation framework that supports short‐target sensing, simultaneous multi‐target detection, and assay designs that separate target recognition from surface anchoring. These greatly expand the application potential of Cas13a in molecular diagnosis and biosensor development.

## 2 Results and Discussion

### 2.1 CRISPR/Cas13a‐CTAM System

By systematically truncating the 28‐nt single‐effect RNA designed by Wang et al. [13] from both its 5' and 3' ends, we constructed a series of double‐effector combinations composed of two short RNA fragments (Table S1), referred to as Tn + Tm'. The core principle behind this design is “functional decoupling”: traditional long‐chain targets are responsible for both stable binding to crRNA and the induction of Cas13a allosteric activation. We hypothesize that these two functions can be carried out by spatially independent short fragments. Specifically, Tn (which targets the 5' end of the crRNA seed‐adjacent region) primarily triggers allostery, while Tm' (which targets the downstream sequence) primarily provides binding affinity and complex stability. The variables n and m represent the number of bases complementary to the crRNA, respectively (Figure S1).

The activation efficiency of these combinations on Cas13a was evaluated by RT‐qPCR (Figure S2, Figure 1b). Notably, when tested as a single effector, Tm′ of all lengths failed to activate Cas13a, whereas cleavage activity increased with increasing Tn. When Tn < 16 nt, Cas13a exhibits only background activity, consistent with Wang et al. [13]. This result likely reflects the proximity of Tn to the crRNA seed‐adjacent region, which is critical for triggering Cas13a allosteric activation. We therefore quantified the signal‐to‐noise ratio for each double‐effector combination based on Tn (black dotted line in Figure 1b). The fluorescence values and signal‐to‐noise ratios exhibited characteristic M‐shaped curves. The intrinsic mechanism behind the M‐shaped curve formation likely arises from competition between the double‐effector during synergistic Cas13a activation: one fragment primarily drives allosteric activation, whereas the other mainly stabilizes the complex. Briefly, when the length of Tn is too short, Cas13a fails to trigger allostery effectively, resulting in insufficient activity. Extending Tn to 12–13 nt enables robust allosteric activation, and a moderately long Tm' can stabilize the complex, producing the first activity peak. As Tn increases further, the available binding space for Tm' is reduced, leading to decreased Cas13a activity. With additional extension, Tn can support both allosteric activation and complex stabilization, giving rise to a second activity peak. Among the tested pairs, T13 + T15', T14 + T14', and T15 + T13' achieved activation efficiencies comparable to the full‐length target T28, indicating that CRISPR/Cas13a‐CTAM can functionally decouple T28 into two independent, editable short RNA fragments while maintaining strong catalytic activity.

To further investigate the synergistic relationship between Tn and Tm', we examined the differences in Cas13a activation caused by single‐base deletions (Figure S3, Figure 1c) and multi‐base continuous deletions (Figure S4, Figure 1d) within the double‐effector combinations. Results revealed that single‐base deletions from the 13th to the 15th position led to a gradual increase in Cas13a's enzymatic activity, with no activity observed when the 13th base was deleted. This suggests that the 13th base is likely to be located near or within the crRNA seed region. This is consistent with Tambe et al.'s model that the seed/mismatch‐sensitive region of Cas13a spans positions 9–14 of the crRNA spacer, as demonstrated through systematic mismatch and binding analysis. However, these conclusions remain inferred and will need confirmation through subsequent structural studies. Deletions from the 16th to 18th bases indicated that contribution of Tm' to Cas13a activation decreased, and the primary factor driving activation was the increasing length of Tn.

The results of the multi‐base continuous deletions showed a gradual reduction in Cas13a activation as the number of missing bases increased. With more than three consecutive base deletions, Cas13a activation was generally lost. Based on fluorescence intensity, the signal‐to‐background ratio (Figure 1), and kinetic analyses (Figure S2), T13 + T15' and T15 + T13' showed the best detection performance under single‐effector RNA conditions. This advantage is manifested as significant synergistic activation effect and extremely low background signal. Performance‐wise, both combinations exhibited high signal‐to‐noise ratios and reached the reaction plateau rapidly, indicating strong detection sensitivity and fast response. Mechanistically, both pairs fall within the peak region of the M‐shaped activity profile, suggesting an optimal balance between allosteric triggering and complex binding stability. This structure–function correspondence not only explains their excellent activation performance but also provides a mechanistic basis for subsequent applications.

### 2.2 Optimization and Performance Evaluation of CRISPR/Cas13a‐CTAM System

**FIGURE 2:**
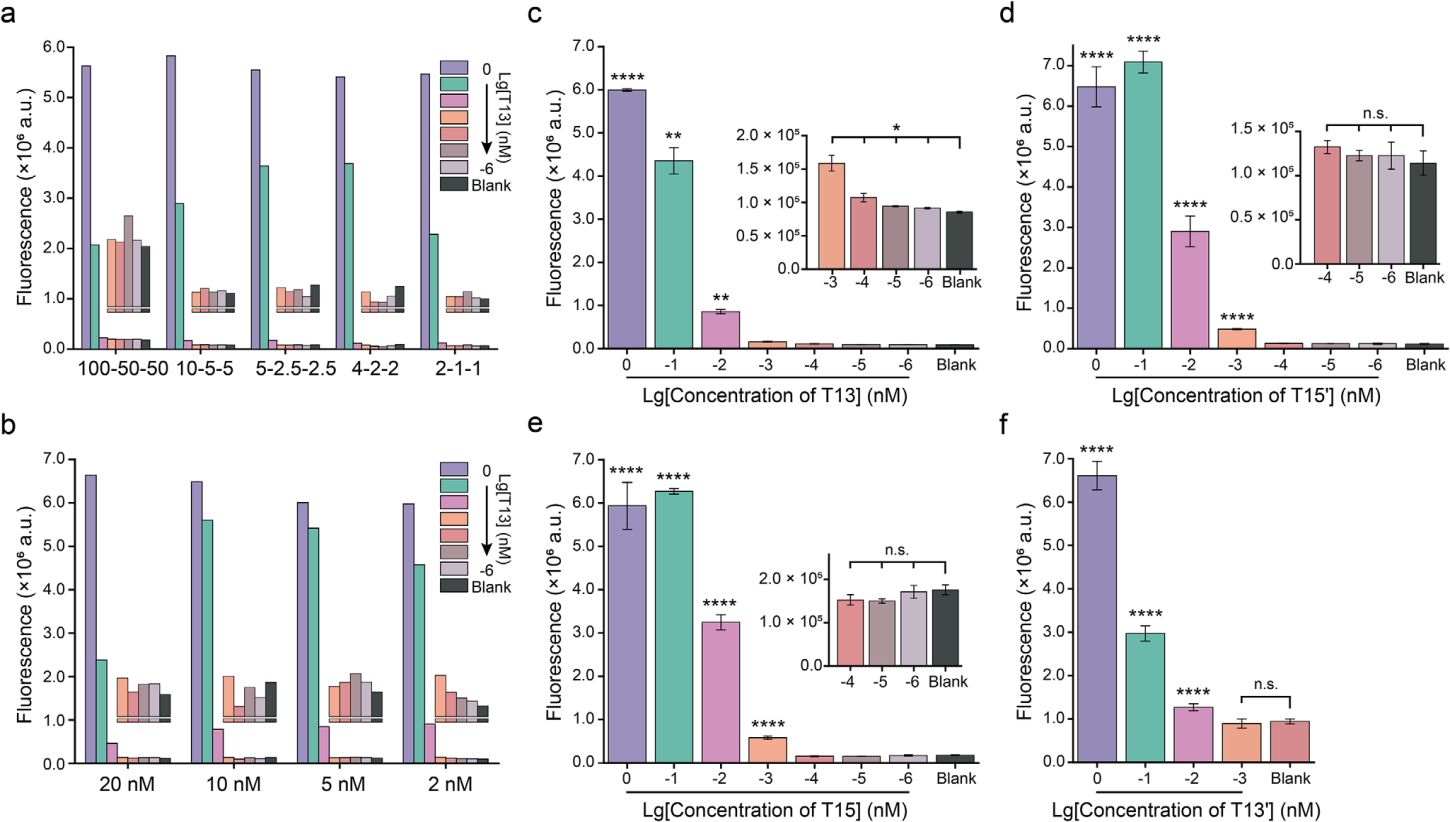
Optimization of the CRISPR/Cas13a‐CTAM system and evaluation of unilateral target detection performance. (a) Optimization of premixed solution concentration. (b) Optimization of T15' concentration. (c) Detection of T13 with fixed T15'. (d) Detection of T15' with fixed T13. (e) Detection of T15 with fixed T13. (f) Detection of T13' with fixed T15. Representative time‐history data are shown in Figures S5–S7. Data are presented as mean ± SD, n = 3, with three independent replicates. Two‐tailed Student's *t*‐tests were used for comparisons, **p < 0.01, ****p < 0.0001.

Given that, during the synergistic activation of Cas13a by the double‐effector combination, two effector RNAs bind independently and randomly to crRNA, Cas13a is activated only when both effector RNAs bind to the same crRNA. Therefore, maximizing the proportion of ternary complexes in the system is crucial. Reducing the concentration difference between crRNA and the target molecule has been considered an effective and feasible approach. The concentration ratio of Cas13a, crRNA, and T15' in the premix was fixed at 2:1:1, and the detection performance of T13 was compared under different premix concentrations. The premix group containing 100 nM Cas13a, 50 nM crRNA, and 50 nM T15' was labeled as 100‐50‐50. After comprehensive comparisons of the time to reach the plateau, background intensity, and sensitivity, the 4‐2‐2 premixed solution was identified as optimal (Figure S5, Figure 2a). However, further increasing the concentration of T15' did not improve the detection performance of T13 (Figure S6, Figure 2b), likely because the binding efficiency between T15' and crRNA was already high at that concentration.

Furthermore, we investigated the detection ability of the T13 + T15' group (Figure S7, Figure 2c,d) and the T15 + T13' group (Figure S7, Figure 2e,f) for unilateral targets in a 4‐2‐2 system. The results showed that the detection sensitivity of the T13 + T15' group for unilateral targets was significantly higher than that of the T15 + T13' group. Specifically, the detection sensitivity of the T13 + T15' group for T13 reached 1 fM, which was lower than the detection sensitivity of Cas13a for traditional single‐effector RNA targets (20–28 nt), which was 10 fM. This increased sensitivity is likely because T13 is closer to the conserved region of crRNA, which coincides with the crRNA seed region, a critical site for Cas13a allosteric activation. Therefore, we conclude that Cas13a is more sensitive to T13 than to the traditional 20–28 nt target. This finding aligns with the conclusion that the 13th base is located near or within the seed region. When CRISPR/Cas13a‐CTAM is used for single‐target detection, the recommended strategy is to fix Tm' with a lower background to detect the more sensitive Tn.

CRISPR/Cas13a‐based single‐nucleotide mismatch analysis in nucleic acids represents a significant area of research [17, 18]. The effect of single‐nucleotide mismatches on CRISPR/Cas13a cleavage activity is complex, influenced not only by the mismatch location but also by the binding affinity between the target and crRNA. Notably, the contribution of binding affinity to enzyme activation has often been overlooked. In contrast to the study by Jennifer et al., which examined the impact of a 20‐nt target with a single‐nucleotide mismatch (T20mN) on CRISPR/Cas13a trans‐cleavage activity [18], we hypothesize that using a shorter 13‐nt target (T13mN) with a mismatch may enhance sensitivity by increasing the influence of binding affinity on CRISPR/Cas13a trans‐cleavage activity.

**FIGURE 3:**
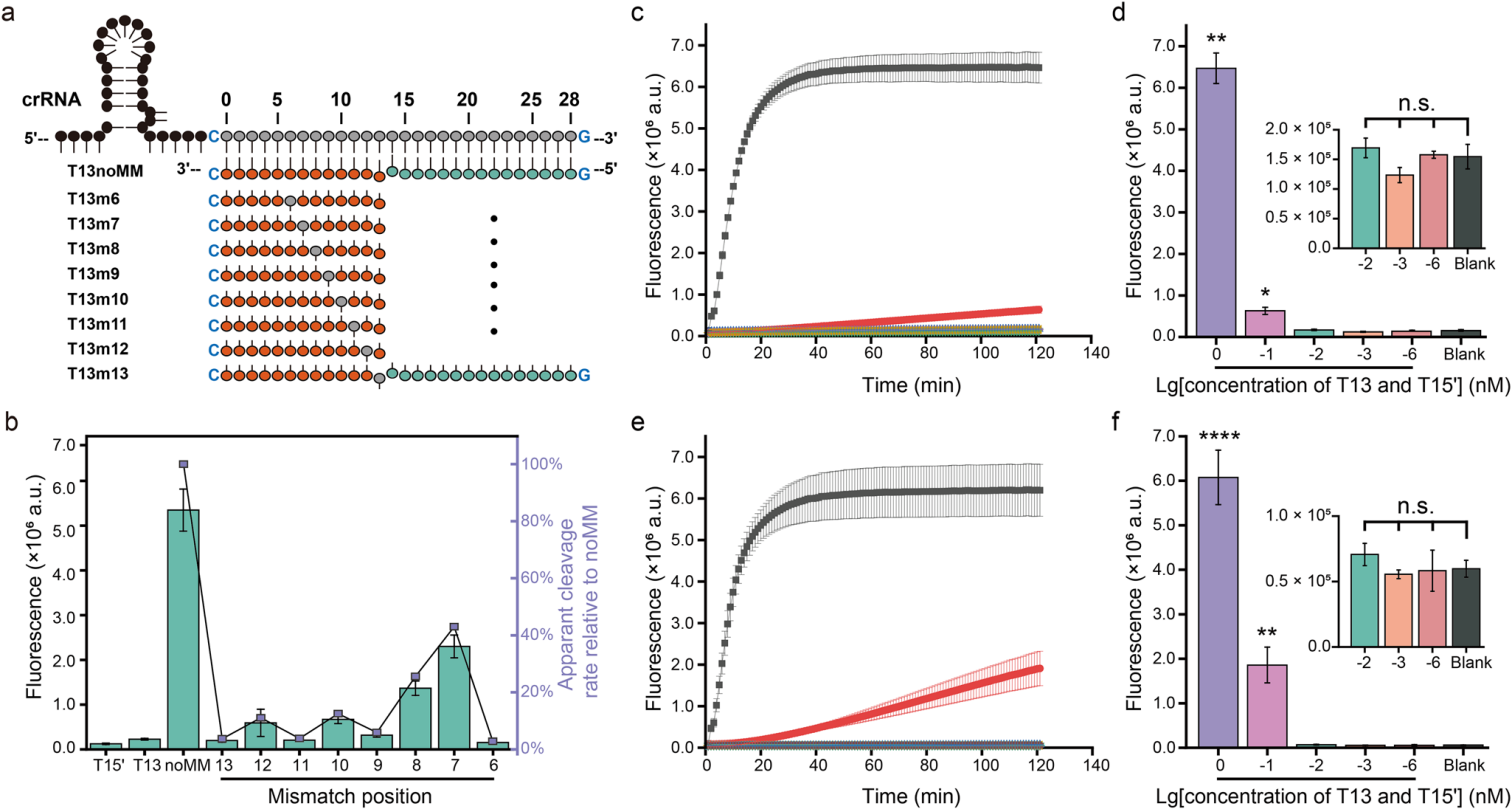
Evaluation of CRISPR/Cas13a‐CTAM Sensitivity to Single‐Nucleotide Mismatches and Dual‐Target Simultaneous Detection Performance. (a) Schematic of single‐nucleotide mismatches in the T13 target (T13mN). (b) The effect of T13mN on Cas13a trans‐cleavage activity was evaluated. Apparent cleavage rates are presented as normalized percentages relative to the no‐mismatch control (noMM). Representative time‐course data are provided in Figure S8. (c) Representative time‐course data for the simultaneous detection of T13 and T15' by CRISPR/Cas13a‐CTAM under 4‐2 conditions. (d) Endpoint fluorescence data from (c) are presented as a histogram. (e,f) Simultaneous dual‐target detection under 0.8–0.4 conditions. Data are presented as mean ± SD, n = 3, with three independent replicates. Two‐tailed Student's *t*‐tests were used for comparisons, *p < 0.05, **p < 0.01, ****p < 0.0001.

As shown in Figure 3a, we designed a series of T13mN targets (Table S1) to assess the sensitivity of the CRISPR/Cas13a‐CTAM system to different mismatches (Figure 3b, Figure S8). The results indicate that T13m6 leads to the most significant defect in enzymatic cleavage activity, consistent with the findings of Jennifer et al. [18] Furthermore, T13m6 caused a 35‐fold reduction in the apparent cleavage rate, a much greater decrease compared to the fivefold reduction observed with T20m6 [18]. Mismatches at positions 9, 11, and 13 also had pronounced negative effects on Cas13a's cleavage activity, with the mismatch at position 13 producing an activity defect similar to base deletion. We propose that the T13 sequence likely contains the seed region of Cas13a. In conclusion, Cas13a exhibits greater sensitivity to shorter T13 sequences. As the target length decreases, the role of binding affinity in modulating cleavage activity becomes more significant, resulting in enhanced sensitivity and resolution for T13mN mismatches. These findings suggest that the CRISPR/Cas13a‐CTAM system could serve as a valuable tool for single‐nucleotide mismatch analysis in short RNA sequences.

Additionally, we investigated the ability of the CRISPR/Cas13a‐CTAM system to simultaneously detect T13 and T15' under 4‐2 conditions (Figure 3c,d). Our findings indicate that the detection remains limited by the random binding of T13 and T15' to crRNA. To enhance detection performance, we propose reducing the concentrations of crRNA and Cas13a (0.8–0.4), though this may reduce the dynamic detection range (Figure 3e,f).

Beyond concentration optimization, several feasible strategies may be considered to further alleviate this probabilistic constraint. One approach involves dynamic and probabilistic control through methods such as fine‐tuning effector‐to‐crRNA ratios, implementing sequential target addition, or applying time‐gated readout strategies to bias the system toward productive ternary complex formation. Alternatively, spatial confinement and local enrichment, achieved via surface immobilization, scaffold‐mediated co‐localization, or compartmentalization within microdroplets or microchambers, could reduce diffusion dimensionality and increase the effective local concentration of effectors around individual crRNA molecules. At a more fundamental level, crRNA or effector engineering may enable cooperative or conditional assembly, thereby transforming stochastic binding into more synergistic interactions. Together, these directions provide realistic paths for improving dual‐target detection performance while remaining compatible with the current CRISPR/Cas13a‐CTAM framework.

### 2.3 CRISPR/Cas13a‐CTAM‐Based Dual miRNA Detection for COVID‐19

Following the validation of CRISPR/Cas13a‐CTAM for short RNA targets under controlled conditions, we next assessed its utility in relevant diagnostic settings. We selected COVID‐19 as a model disease, motivated by the pressing need for accurate and multiplexed RNA detection methods. Specifically, we targeted two circulating miRNAs—miR‐155 and miR‐499, which have been independently reported to be upregulated in COVID‐19 patients [19]. Their simultaneous detection offers a potential strategy to enhance diagnostic specificity and to capture distinct pathological features of the disease.

**FIGURE 4:**
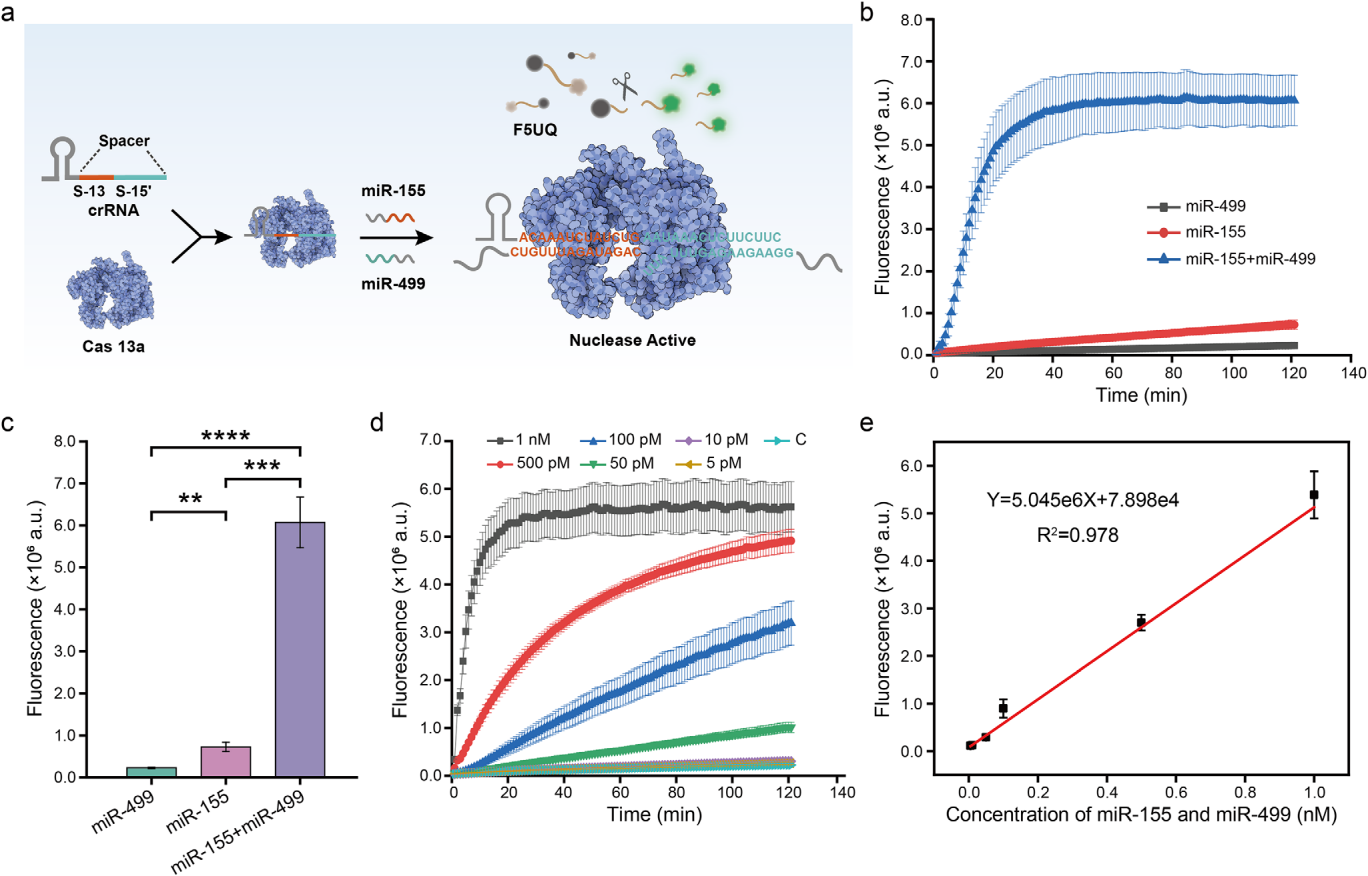
CRISPR/Cas13a‐CTAM‐based dual miRNA detection for COVID‐19. (a) Schematic representation of CRISPR/Cas13a‐CTAM‐based dual miRNA detection for COVID‐19. (b,c) Feasibility assessment of miR‐155 and miR‐499 in coordinating Cas13a activation. (d) Representative time‐course curves in the absence of dual RNA activators and in the presence of varying concentrations of T13 and T15'. (e) Correlation curve illustrating the relationship between fluorescence intensity at 30 min from (d) and the concentration of dual targets. Data are presented as mean ± SD, n = 3, with three independent replicates. Two‐tailed Student's t tests were used for comparisons, **p < 0.01, ***p < 0.001, ****p < 0.0001.

By designing crRNA spacer regions complementary to the 3' end of miR‐155 and the 5' end of miR‐499 with 13 and 15 bases, respectively (Figure 4a). Results demonstrated the feasibility of co‐activating Cas13a using these two miRNAs (Figure 4b,c). Additionally, the sensitivity of CRISPR/Cas13a‐CTAM for the dual detection of miR‐155 and miR‐499 was evaluated. Figure 4d shows representative time‐course curves in the absence of dual RNA activator and in the presence of varying concentrations of T13 and T15' (5–1000 pM). Using direct fluorescence readout, the CRISPR/Cas13a‐CTAM system can achieve dual miRNA detection at concentrations as low as 15 pM (*S*/*N* = 3) (Figure 4e).

### 2.4 CRISPR/Cas13a‐CTAM‐Based Combined Detection of Exosome Membrane Proteins

After establishing the high specificity and versatility of CRISPR/Cas13a‐CTAM for dual RNA targets in solution, we next evaluated its performance in a more complex, biologically relevant setting. To this end, we adapted the system for the multiplexed detection of protein biomarkers on the surface of exosomes, which are key targets in liquid biopsy. This extension aimed to underscore that CRISPR/Cas13a‐CTAM is a modular, programmable diagnostic platform with broad applicability that extends beyond nucleic‐acid targets.

**FIGURE 5:**
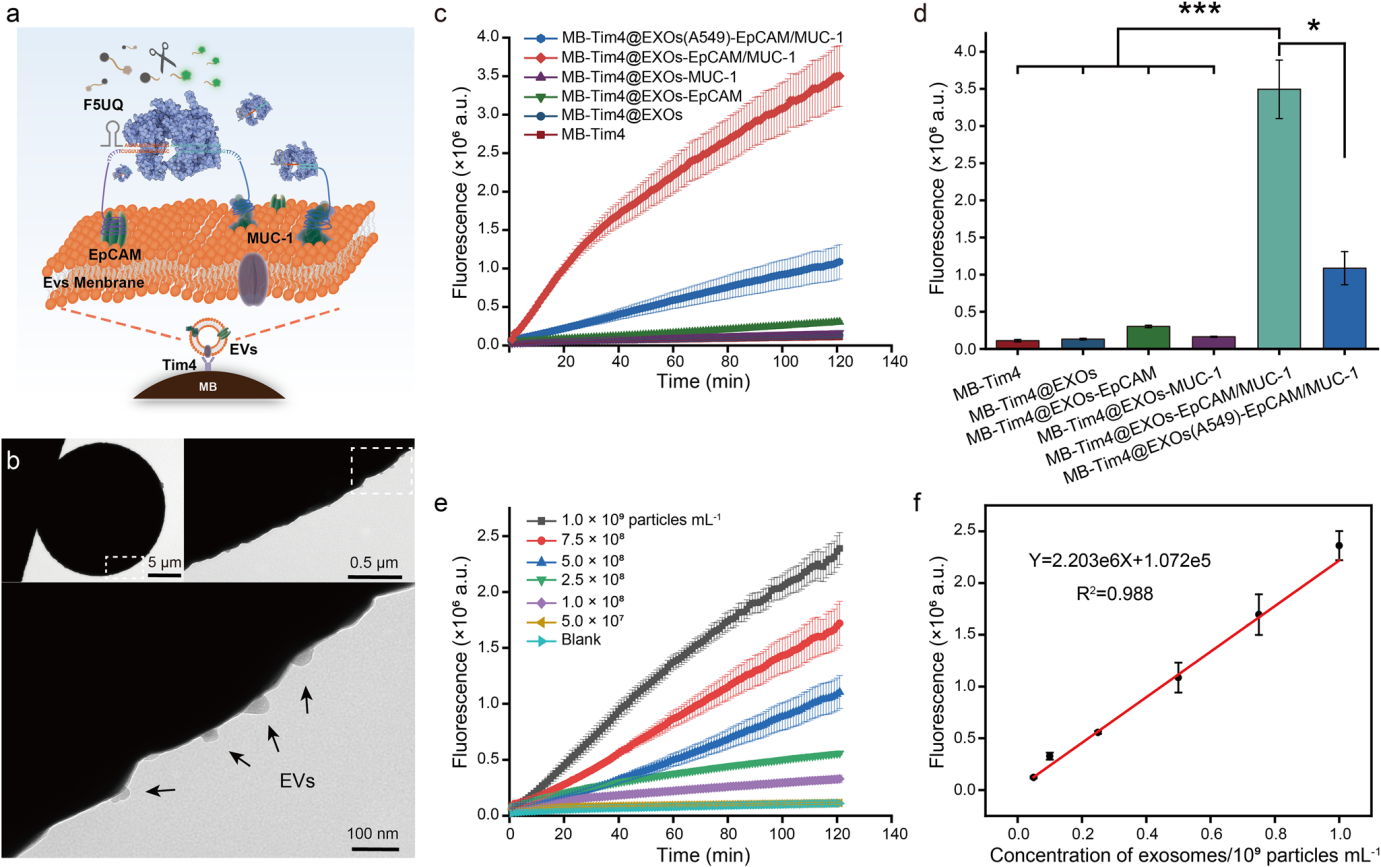
CRISPR/Cas13a‐CTAM‐based combined detection of exosome membrane proteins. (a) Schematic representation of CRISPR/Cas13a‐CTAM‐based combined detection of exosome membrane proteins. (b) The enrichment of exosomes on the surface of Tim4 functionalized magnetic beads was characterized by TEM. (c, d) Feasibility assessment of CRISPR/Cas13a‐CTAM‐based combined detection of exosome membrane proteins. (e) Representative time course curves for the exosomes detection at various concentrations. (f) Correlation curve illustrating the relationship between endpoint fluorescence intensity from (d) and exosome concentration. Data are presented as mean ± SD, n = 3, with three independent replicates. Two‐tailed Student's *t*‐tests were used for comparisons, ***p < 0.001.

Exosomes, ubiquitous nanoscale endocrine vesicles, are promising early tumor markers due to their close association with parental cells [20, 21, 22]. However, the accuracy of cancer detection based on single exosome membrane proteins remains suboptimal due to the lack of highly specific tumor‐derived exosome markers. Thus, the combined detection of exosome membrane proteins offers a more effective and precise approach for distinguishing and quantifying tumor‐derived exosomes. The CRISPR/Cas13a‐CTAM system is well‐suited to address this issue, and we applied it to the combined detection of MCF‐7‐derived exosome membrane proteins EpCAM and MUC‐1 (Figure 5a). EpCAM and MUC‐1 are known to be highly expressed on the surface of MCF‐7‐derived exosomes. First, Tim4‐functionalized magnetic beads were used to bind specifically to the phosphatidylserine (PS) abundant on the exosome membrane, facilitated by calcium ions, enabling exosome capture and enrichment [23, 24, 25]. Next, EpCAM and MUC‐1 aptamer sequences were concatenated to the 3' end of T13 (Apt‐T13) and the 5' end of T15' (Apt‐T15'), respectively. Apt‐T13 and Apt‐T15' were co‐incubated with Tim4‐enriched exosomes. Apt‐T13 and Apt‐T15' were co‐incubated with Tim4‐enriched exosomes. Cas13a activation occurred only when MUC‐1 [26] and EpCAM [27] proteins were both highly expressed on the exosome surface, mediated by the synergistic action of Apt‐T13 and Apt‐T15'. Finally, the concentration of target exosomes was quantified by directly measuring fluorescence intensity.

First, exosomes purified by ultracentrifugation were characterized using nanoparticle tracking analysis (NTA) and transmission electron microscopy (TEM) (Figure S9). These exosomes were then incubated with Tim4‐functionalized magnetic beads. TEM imaging confirmed the successful enrichment of exosomes on the surface of the magnetic beads (Figure 5b). To further evaluate assay specificity, exosomes derived from the A549 cell line were introduced as a negative control, as previous studies have reported relatively high EpCAM expression but low MUC‐1 abundance on A549‐derived exosomes. As shown in Figure 5c,d, only MCF‐7‐derived exosomes induced strong Cas13a activation, whereas the signal obtained from A549‐derived exosomes was reduced to approximately one‐quarter of that observed for MCF‐7. These results demonstrate that high expression of a single antigen is insufficient to efficiently activate Cas13a, confirming the dual‐input requirement of the CRISPR/Cas13a‐CTAM‐based assay. Finally, the sensitivity of the CRISPR/Cas13a‐CTAM system for the combined detection of adjacent proteins on the exosome membrane surface was evaluated. The fluorescence intensity exhibited a strong linear relationship with exosome concentration in the range of 5.0 × 10^7^ to 1.0 × 10^9^ particles mL^−1^, with a detection limit as low as 2.4 × 10^7^ particles mL^−1^ (*S*/*N* = 3).

It should be noted that the synergistic activation observed here does not require EpCAM and MUC‐1 to be in immediate nanoscale contact on the exosome membrane. Both Apt‐T13 and Apt‐T15' contain a flexible thymine‐rich linker, which provides substantial conformational freedom and allows the aptamer–RNA constructs to accommodate variations in inter‐protein spacing. In addition, given the small diameter of exosomes (typically ∼50–150 nm), the effective local concentrations of surface proteins are intrinsically high. Within this confined membrane area, dynamic repositioning of the flexible aptamer–RNA conjugates enable efficient sampling of the exosome surface, thereby increasing the probability that the two aptamer‑tethered effectors can cooperatively assemble and synergistically activate Cas13a.

### 2.5 CRISPR/Cas13a‐CTAM‐Based Immobilization of Cas13a

**FIGURE 6:**
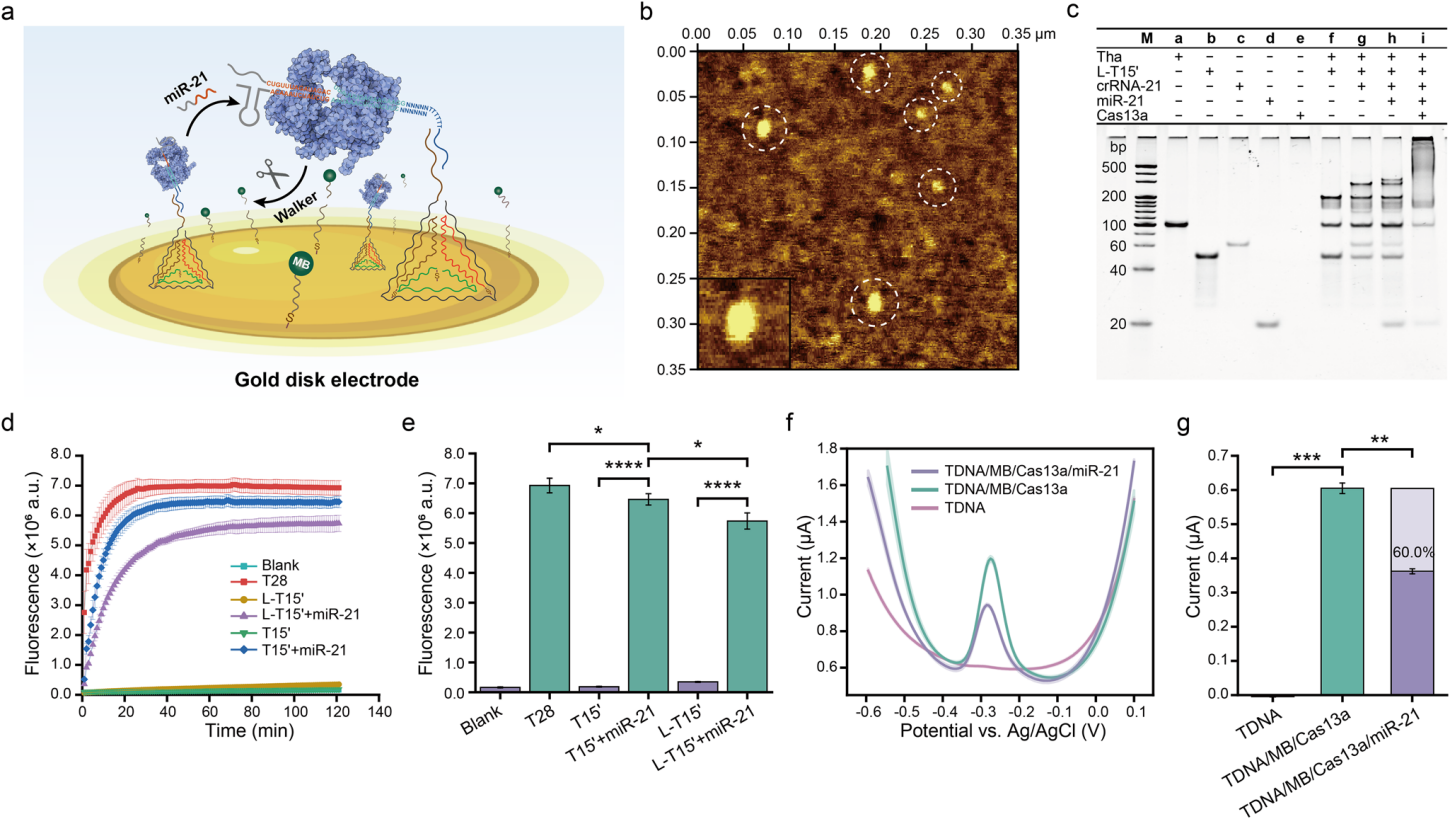
CRISPR/Cas13a‐CTAM‐based Cas13a immobilization for miRNA electrochemical detection. (a) Schematic diagram. (b) The nanomorphology of TDNA was characterized using AFM. (c) Sequential assembly of THa, L‐T15', crRNA, miR‐21, and Cas13a was confirmed by 12.5% PAGE. (d,e) Comparison of Cas13a activation between T15' and L‐T15'. (f,g) Demonstration of miRNA‐21 detection feasibility using a Cas13a‐functionalized electrode. Data are presented as mean ± SD, n = 3, with three independent replicates. Two‐tailed Student's *t*‐tests were used for comparisons, *p < 0.05, **p < 0.01, ***p < 0.001, ****p < 0.0001.

To demonstrate the adaptability of CRISPR/Cas13a‐CTAM beyond homogeneous assays, we leveraged its dual‐effector design to achieve the first functional immobilization of Cas13a on a solid substrate. Since T15' barely activates Cas13a, we utilized T15' as an anchoring sequence to functionalize inactive Cas13a on a material surface, thereby broadening its application potential. As a demonstration, we present in situ electrochemical detection of miRNA using a Cas13a‐functionalized electrode (Figure 6a). In this approach, a thiolated DNA tetrahedron (TDNA) with an extended sequence is anchored to the gold disk electrode surface via an Au‐S bond [28, 29, 30]. L‐T15', which contains six additional bases complementary to crRNA compared to T15', serves as a linker, binding to the 3' end of crRNA and the 5' end of the TDNA extension sequence to immobilize Cas13a on the electrode surface. When the target miRNA‐21 is present, it cooperates with L‐T15' to activate Cas13a's trans‐cleavage activity, resulting in the cleavage and dissociation of the MB probe modified on the electrode surface. The resulting change in the electrical signal due to the detachment of the MB from the electrode is recorded using square wave voltammetry (SWV).

First, TCEP pretreatment is essential for reducing the disulfide bond in the TDNA monomer (Figure S10a). The assembly of TDNA was confirmed by 12.5% PAGE (Figure S10b), and its microscopic morphology was characterized using AFM (Figure 6b). Additionally, 12.5% PAGE verified the sequential assembly of THa, L‐T15', crRNA, miR‐21, and Cas13a, showing that the addition of Cas13a significantly enhanced the assembly efficiency of the other components (Figure 6c). Fluorescence analysis revealed that compared to T15', the activation effect of L‐T15' on Cas13a decreased to 88.8%, which remained within the acceptable range (Figure 6d,e). This provides further evidence supporting the feasibility of using Tm' to immobilize inactive Cas13a on the sensor surface.

The pre‐treated gold electrode was initially incubated with TCEP‐treated TDNA [30], followed by incubation with the thiolated MB probe. MCH was then applied to block non‐specific adsorption and promote the directional arrangement of the probes. Subsequently, the ternary complex of L‐T15', crRNA, and Cas13a was incubated with the electrode to complete the construction of the Cas13a‐functionalized electrochemical sensor. The results are presented in Figure 6f,g. In the presence of miRNA‐21, a decrease in peak current was observed, attributed to the release of the MB probe from the electrode surface due to Cas13a activation. Based on the charge change of the electrode surface SH‐MB reporter before and after Cas13a activation, we estimated the surface coverage of active Cas13a to be approximately 6.12 × 10^10^ molecules cm^−2^. This quantitative estimate confirms the successful and functional immobilization of Cas13a at a relevant density for sensing applications. These results confirm the feasibility of the CRISPR/Cas13a‐CTAM system for functionalizing Cas13a on material surfaces, paving the way for broader application scenarios.

To evaluate the practical reliability of the CRISPR/Cas13a‐CTAM‐based electrochemical sensing platform, we assessed its intra‐assay and inter‐assay reproducibility (Table S5). Two independent batches of sensors were tested with miRNA‐21. The coefficients of variation (CV) within Batch 1 and Batch 2 were 9.61% and 8.45%, respectively (n = 5), indicating good repeatability within each fabrication batch. The CV calculated from the means of the two batches was 11.70%, demonstrating acceptable consistency between different fabrication batches. These values are within the typical acceptable range for electrochemical biosensors and support the reliability of our sensor for miRNA detection.

## 3 Conclusions

In this work, we introduce CRISPR/Cas13a‐CTAM, a compensatory target activation mechanism that enables synergistic Cas13a activation using two independently programmable short RNA effectors. This dual‐effector RNA architecture preserves cleavage activity comparable to conventional single‐effector formats while markedly expanding design flexibility and application potential. Notably, CRISPR/Cas13a‐CTAM supports single‐nucleotide discrimination, detection of ultra‐short RNA targets, simultaneous dual‐target analysis, and solid‐surface immobilization of Cas13a, thereby addressing key limitations of traditional CRISPR/Cas13a‐based diagnostics.

Importantly, the compensatory target activation mechanism of CRISPR/Cas13a‐CTAM is fundamentally distinct from previously reported Gemini‐like strategies, which rely on the higher‐order assembly of two Cas13a: crRNA complexes to achieve dual‐input sensing. In contrast, CTAM operates within a single Cas13a effector by functionally decoupling allosteric triggering and binding stabilization into two independently programmable short RNA fragments. This simpler activation logic reduces structural complexity while providing enhanced modularity for short‐target recognition and surface‐integrated biosensor development.

We first evaluated the ability of different double‐effector combinations to activate Cas13a. Our results demonstrated that Tn is essential for Cas13a allosteric activation, whereas Tm' contributes comparatively little. This finding is consistent with previous reports indicating that the Tn sequence contains the Cas13a seed region. Single‐base mismatch experiments further indicated that the seed region spans 9–13 nt. We therefore hypothesize that Tn primarily drives allosteric activation, whereas Tm' mainly stabilizes the Cas13a–target RNA complex. The optimal combination, T13 + T15', exhibited a cooperative, all‐or‐nothing activation profile, which we attribute to an optimal balance between allosteric activation and complex stability. Importantly, the functional decoupling of single‐effect RNA by CRISPR/Cas13a‐CTAM greatly improves its sensitivity to short RNA sequences. This enhanced sensitivity was reflected in two aspects: first, the detection limit for short RNA targets decreased to 1 fM for the T13 target, representing a tenfold improvement over wild‐type Cas13a; second, CRISPR/Cas13a‐CTAM exhibited increased sensitivity to single‐base mismatches. The T13m6 mismatch reduced the apparent cleavage rate by 35‐fold, an effect seven times greater than that observed for the T20m6 mismatch.

Beyond sensitivity gains, CRISPR/Cas13a‐CTAM intrinsically enables multiplexed sensing through coordination of two effectors: dual miRNA profiling for COVID‐19–associated biomarkers (miR‐155 and miR‐499) and combined detection of exosomal membrane proteins (EpCAM and MUC‐1) for cancer diagnostics. Coordinating two independent (and separately programmable) RNA effectors simplifies signal‐transduction logic and can enhance specificity via dual‐input requirements, supporting the use of CRISPR/Cas13a‐CTAM as a versatile platform for multiplexed biosensing and molecular diagnostics.

A pivotal technological advance of this work is the successful immobilization of Cas13a on a sensor surface. By using the weakly activating Tm'‐derived sequence as an anchoring module, we immobilized inactive Cas13a on an electrode and achieved in situ electrochemical miRNA detection, bridging CRISPR‐based recognition with functional biointerfaces and supporting integrated biosensor development.

Looking forward, the CRISPR/Cas13a‐CTAM framework holds promise for synergistic integration with other technologies. Its programmable, dual‐input architecture could be integrated with isothermal amplification to further enhance sensitivity, coupled with microfluidics to enable portable point‐of‐care devices, or combined with emerging nanomaterials to support multiplexed spatial imaging. The independent programmability of the two effector RNAs also makes the system a modular component for synthetic biology circuits, enabling logic‐guided molecular diagnostics and adaptable target design.

In addition to functional integration with amplification, microfluidics, and synthetic biology circuits, practical considerations such as sensor reusability and long‐term storage stability will be critical for future translational applications of the CRISPR/Cas13a‐CTAM framework. As an enzyme‐based system, Cas13a activity is expected to gradually decline upon repeated use or prolonged storage under ambient conditions. Future designs could therefore exploit the inherent modularity of the CTAM architecture to enable interface regeneration, for example by decoupling the surface‐anchoring scaffold from the Cas13a‐binding activator and introducing strand‐displacement strategies to refresh the functional ternary complex on demand. For long‐term preservation, an alternative operational mode may involve storing the sensor in a pre‐functionalized state with only the anchoring scaffold immobilized on the interface, while introducing Cas13a immediately prior to use. Owing to the high specificity of the dual‐effector activation mechanism, free Cas13a does not generate significant background signals, which may relax washing requirements and simplify practical operation. Together, these considerations highlight the potential of the CRISPR/Cas13a‐CTAM system to evolve from a single‐use analytical platform into a more robust and adaptable sensing technology through rational interface and workflow design.

Collectively, CRISPR/Cas13a‐CTAM provides a robust and adaptable framework that expands the utility of CRISPR/Cas13a in RNA diagnostics, multiplexed detection, and sensor integration, laying a strong foundation for precise, flexible, and field‐deployable molecular detection tools.

## 4 Experimental Section

### 4.1 Regent and Instruments

All nucleic acid sequences and probes were ordered from Hema Biotechnology Co., Ltd. (Huzhou, China). Real‐time quantitative polymerase chain reaction (RT‐qPCR) was conducted using the QuantStudio 3 system from ThermoFisher. Exosome purification was performed with the Optima MAX‐XP ultracentrifuge (Beckman Coulter). The electrochemical experiments were conducted on the CHI660e electrochemical workstation from Chenhua Instrument Co., Ltd. (Shanghai, China).

### 4.2 LbuCas13a Expression and Purification

LbuCas13a was expressed and purified from *Escherichia coli* BL21(DE3) containing the expression vectors obtained from Addgene (Plasmid #83482). The detailed experimental procedures are available in Shan and Zhao et al. [31, 32].

### 4.3 CRISPR/Cas13a‐CTAM RT‐qPCR Reaction System

For each 10 µL RT‐qPCR reaction, crRNA, Cas13a, 10× CutSmart Buffer (New England Biolabs), Tn, Tm', and FQ5U each comprised 10% of the total volume. The FQ5U sequence is FAM‐UUUUU‐BHQ1, a fluorescent reporter probe designed based on Cas13a's preference for U bases during trans‐cleavage activity. DNase/RNase‐free water (TakaRa) was used to adjust the volume in the absence of target components. The molar ratio of Cas13a to crRNA was maintained at 2:1, and the concentrations of Cas13a, crRNA, target, and FQ5U were 100 nM, 50 nM, 1 nM, and 2 µM, respectively, unless otherwise specified. To minimize Cas13a's exposure to the environment, crRNA, CutSmart Buffer, and DNase/RNase‐free water were mixed first, followed by the addition of Cas13a. Subsequently, 7 µL of the premixed solution was added to 0.2 mL DNase/RNase‐free PCR eight‐tube strips (Sangon). Then, 1 µL of target and FQ5U were added sequentially, ensuring they adhered to the tube wall. After a 5‐s centrifugation, the samples were immediately placed in the RT‐qPCR instrument for detection, with readings taken every minute for 120 min.

### 4.4 Cell Culture and EVs Isolation

MCF‐7, a human breast cancer cell line obtained from the National Collection of Authenticated Cell Cultures (Shanghai, China), was selected for testing. All cell lines were screened for mycoplasma contamination to ensure quality. Cells were cultured in T25 flasks and expanded to 8 flasks to facilitate the collection of extracellular vesicles [33]. Cells were grown and passaged in RPMI‐1640 medium supplemented with 10% FBS and 1% penicillin/streptomycin (Gibco) at 37°C with 5% CO_2_.

### 4.5 EVs Isolation and Characterization

To induce cellular starvation, the medium was replaced with RPMI‐1640 lacking FBS and supplemented with 1% penicillin/streptomycin when the cells reached approximately 80% confluency. After 40 h, the supernatant was collected for further processing. The supernatant was sequentially centrifuged at 300× g, 2000× g, and 10 000× g for 10, 10, and 30 min, respectively, at 4°C to remove cells and debris. The resulting supernatant was filtered through a 0.22 µm pore filter and subjected to ultracentrifugation at 100 000× g for 75 min at 4°C using a Beckman ultracentrifuge to pellet the EVs. The pellet was resuspended in 10 mL PBS, followed by a second ultracentrifugation at 100 000× g for 75 min at 4°C, and then resuspended in 600 µL PBS. The final EV suspension was stored at −80°C, with a recommended storage duration of no more than 3 months for optimal quality. The concentration of exosomes isolated according to the above criteria was 6.30 × 10^10^ particles mL^−1^, and the average particle size was approximately 137.5 nm, which represents the mean value based on three independent NTA (Malvern NanoSight NS300) measurements (Figure S9a). The morphology of the isolated exosomes was further confirmed by TEM to be characteristic of a typical cup‐shaped structure (Figure S9b).

### 4.6 Construction of Tim4 Functionalized Magnetic Beads to Capture Exosomes

A total of 100 µL of streptavidin‐modified magnetic beads (Bangs Laboratories) were washed twice with 100 µL of PS buffer (Sangon) and placed on a magnetic frame for 5 min each time to collect the beads. Next, a mixture of 99 µL of PS buffer and 1 µL of 500 nM biomodified Tim4 (Xinbosheng Biotechnology Co., Ltd., Shenzhen, China) was added to the beads, which were gently stirred at 4°C for 1 h. After incubation, the beads were washed with PS buffer and resuspended in 100 µL of PS buffer. The Tim4‐functionalized magnetic beads were stored at 4°C for no longer than 2 weeks.

### 4.7 Pretreatment of Au Electrode

The 3 mm gold disc electrode (Chenhua Instrument Co., Ltd., Shanghai, China) was first immersed in a freshly prepared piranha solution for 30 min, after which it was cleaned by ultrasonication in anhydrous ethanol and deionized water for 3 min. Next, the electrode was polished on a suede pad with 0.3 µm and 0.05 µm Al_2_O_3_ powder until it appeared mirror‐like. Following another ultrasonic cleaning step, the electrode was then subjected to cyclic voltammetry in freshly prepared 0.5 M sulfuric acid until a stable curve was obtained, with a platinum electrode serving as auxiliary and a saturated Ag/AgCl electrode serving as reference. The electrochemical workstation CHI660e was purchased from Chenhua Instrument Co., Ltd in Shanghai, China.

### 4.8 Pretreatment and Assembly of TDNA

TDNA monomers were pretreated with 3 mM TCEP (Sigma) at room temperature for 2 h to reduce disulfide bonds. The mixture was then heated to 95°C for 10 min in a PCR instrument and rapidly cooled in an ice‐water bath to form a stable tetrahedral structure. TDNA can be stored at 4°C for up to 1 week.

### 4.9 Atomic Force Microscopy Characterization of TDNA

First, 40 µL of freshly prepared 0.5% (wt/vol) APTES in anhydrous ethanol was applied to the center of a freshly cleaved mica surface and incubated for 2 min. The surface was then rinsed thoroughly with deionized water to remove excess APTES and dried under nitrogen. Next, 20 µL of freshly prepared 100 nM TDNA was deposited onto the treated mica surface. Following a 15‐minute incubation, 80 µL of TM buffer (50 mM Tris‐HCl, 10 mM magnesium sulfate, pH 7.5) was added. Imaging was conducted using the liquid‐phase tapping mode of the Cypher ES atomic force microscope (AFM) at a scanning speed of 2.44 Hz, utilizing the BLAC‐40 probe.

### 4.10 Gel Electrophoresis Analysis

A 12.5% polyacrylamide gel electrophoresis (PAGE) was used for analysis, with 1× TBE buffer and Gel Red as the oligonucleotide stains. THa, L‐T15', crRNA, miR‐21, and Cas13a, as well as the sequentially assembled complexes, were pre‐incubated in 1× PBS at 25°C for 10 min, then mixed with loading buffer. Each lane was loaded with 6 µL of the sample complex and subjected to electrophoresis at 120 V for 45 min at room temperature. Gel images were captured using the Gel Doc XR+ imaging system (Bio‐Rad).

### 4.11 Construction of the Cas13a‐Functionalized Sensor and Detection of miRNA‐21

The pretreated gold electrode was immersed in a freshly prepared 1 µM TDNA solution and incubated in the dark at room temperature for 2 h. After rinsing with sterilized deionized water, it was incubated with 1 µM TCEP‐treated MB probe for 2 h. Surface non‐specific adsorption sites were blocked by incubation with 1 mM MCH for 1 h. The electrode was then incubated with 1× PBS premix containing 100 nM Cas13a, 50 nM crRNA‐21, and 50 nM L‐T15' for 10 min. Following gentle washing with PBS, the electrode was incubated with miRNA‐21 for 15 min. Square wave voltammetry (SWV) was used to record the signal, with a voltage range of −0.6 to 0.1 V, a step size of 5 mV, an amplitude of 25 mV, and a frequency of 25 Hz. Unless otherwise stated, all reagents used in this section were RNase‐free or DCEP‐treated.

### 4.12 Electrochemical Detection Stability and Reproducibility

To evaluate the reliability and consistency of the CRISPR/Cas13a‐CTAM‐based electrochemical sensor, intra‐assay and inter‐assay reproducibility tests were conducted using miRNA‐21 as the model target. For intra‐assay reproducibility, two independent batches of sensors were fabricated following the same procedure described above. Each batch consisted of five replicate sensors. The peak current responses were measured using SWV under identical conditions.

### 4.13 Calculation of Cas13a Coverage and Immobilization Efficiency

Based on our available data, we can estimate the surface coverage and immobilization efficiency of Cas13a as follows:

(i) Assumptions and experimental basis. We assume a uniform distribution of thiolated methylene‑blue (SH‑MB) probes on the gold‑disk electrode (radius R). The change in charge associated with the SH‑MB signal before and after Cas13a‑mediated cleavage, measured by SWV, reflects the proportion of the electrode area that falls within the effective activity range of surface‑tethered Cas13a. The fractional signal decrease *k* is calculated from the SWV peak areas:

$$k=\frac{S_{0}-S_{1}}{S_{0}}$$

where *S*_0_ and *S*_1_ are the integrated peak areas before and after cleavage, respectively.

(ii) Effective activity radius of a single Cas13a molecule. In the design, it can be considered that a total of 50 nucleotides from the first base of the crRNA spacer to the first base of the THa sequence extending on the TDNA is the radius that limits the activity of Cas13 a. Using a standard base distance of 0.34 nm, the effective active radius *r* is approximately:

r≈50×0.34nm=17nm

(iii) Calculation of Cas13a surface coverage. If each Cas13a molecule cleaves all SH‑MB probes within a circular area of radius r, the total number of Cas13a molecules on the electrode, *N*_*Cas*13*a*_ can be estimated as:

$$N_{Cas13a}=\frac{k\cdot \pi R^{2}}{\pi r^{2}}$$

Substituting the experimental values yields:

$$N_{Cas13a}=7.69\times 10^{9}\;\mathrm{molecules}$$

The corresponding surface coverage Γ_*Cas*13*a*_ is:

$$\Gamma_{Cas13a}=\frac{N_{cas13a}}{\pi R^{2}}=6.12\times 10^{10}\frac{\mathrm{molecules}}{cm^{2}}$$

(iv) Immobilization efficiency. Using the Cas13a incubation conditions applied in this work (100 nM, 20 µL), the total number of Cas13a molecules offered to the surface is:

$$N_{total}=c_{Cas13a}\times V\times N_{a}=1.2\times 10^{12}\;\mathrm{molecules}$$

Thus, the immobilization efficiency η_*Cas*13*a*_ is:

$$\eta_{Cas13a}=\frac{N_{Cas13a}}{N_{total}}\times 100\%=0.64\%$$

It should be noted that this calculation relies on several simplifying assumptions—including uniform probe distribution, a perfectly circular activity zone for each Cas13a molecule, and complete cleavage within that zone. Furthermore, this study did not systematically optimize parameters that may influence immobilization efficiency (e.g., concentration, volume, incubation time, surface area of anchor sites). Therefore, the above values represent a preliminary estimate under the specific experimental conditions used.

### 4.14 Statistics and Reproducibility

Statistical tests were performed using GraphPad Prism version 8.0, IBM SPSS Statistics version 19.0 and Two‐tailed Student's *t*‐test. The statistical test used for the data shown in each figure is noted in the corresponding figure legend. Significance levels are denoted as follows: *p < 0.05, **p < 0.01, ***p < 0.001, ****p < 0.0001. Data are presented as the mean ± standard deviation (SD), with n = 3 and three independent replicates. No data were excluded from the analyses.

## Author Contributions

J.H. and Y.S. directed the project. J.H. and B.J. conceived the idea. B.J., T.Z., Y.L., S.Z., W.X. carried out the experiments. B.J., J.H. analyzed the data and wrote the paper. J.H., Y.S., S.P., N.S. assisted with data interpretation, commented and revised the paper.

## Conflicts of Interest

The authors declare no conflicts of interest.

## Supporting information




**Supporting File**: advs74968‐sup‐0001‐SuppMat.docx.

## Data Availability

The data that support the findings of this study are available from the corresponding author upon reasonable request.;
