# Supplementary material for: CRISPR/Cas13a: Compensatory Target Activation Mechanism
Source: Adv Sci (Weinh). 2026 Apr 9;13(32):e24156. doi: 10.1002/advs.202524156 (PMC13252659; doi:10.1002/advs.202524156)

*Supplementary Information*

**CRISPR/Cas13a: Compensatory Target Activation Mechanism**

Bowen Jiang^1,2^, Tenghua Zhang^1,2^, Yao Lu^1,2^, Sujun Zhou^2^, Wenjing Xiao^2^, Shuming Pan^4^, Nan Shi^4,^*, Yan Sheng^2,3,^*, Jiaming Hu^1,2,^*

^1^International Joint Laboratory of Catalytic Chemistry, Innovation Institute of Carbon Neutrality, Department of Chemistry, College of Sciences, Shanghai University, Shanghai 200444, China.

^2^MOE Key Laboratory of Laser Life Science & Institute of Laser Life Science, Guangdong Provincial Key Laboratory of Laser Life Science, College of Biophotonics, South China Normal University, Guangzhou 510631, China.

^3^Institute of Translational Medicine, Shanghai University, Shanghai 200444, China.

^4^Emergency Department, Putuo Hospital, Shanghai University of Traditional Chinese Medicine, Shanghai 200062, China.

* Corresponding author.

To whom correspondence should be addressed: ysheng@shu.edu.cn (Y.S.), jmhu@shu.edu.cn (J.H.)

**Table of Contents**

[SUPPLEMENTARY FIGURES 3](#_Toc221586923)

[Figure S1 3](#_Toc221586924)

[Figure S2. 3](#_Toc221586925)

[Figure S3. 4](#_Toc221586926)

[Figure S4. 4](#_Toc221586927)

[Figure S5. 5](#_Toc221586928)

[Figure S6. 6](#_Toc221586929)

[Figure S7. 7](#_Toc221586930)

[Figure S8. 8](#_Toc221586931)

[Figure S9. 8](#_Toc221586932)

[Figure S10 9](#_Toc221586933)

[SUPPLEMENTARY TABLES 10](#_Toc221586934)

[Table S1. 10](#_Toc221586935)

[Table S2. 11](#_Toc221586936)

[Table S3. 11](#_Toc221586937)

[Table S4. 12](#_Toc221586938)

[Table S5. 12](#_Toc221586939)

# ****SUPPLEMENTARY FIGURES****

**
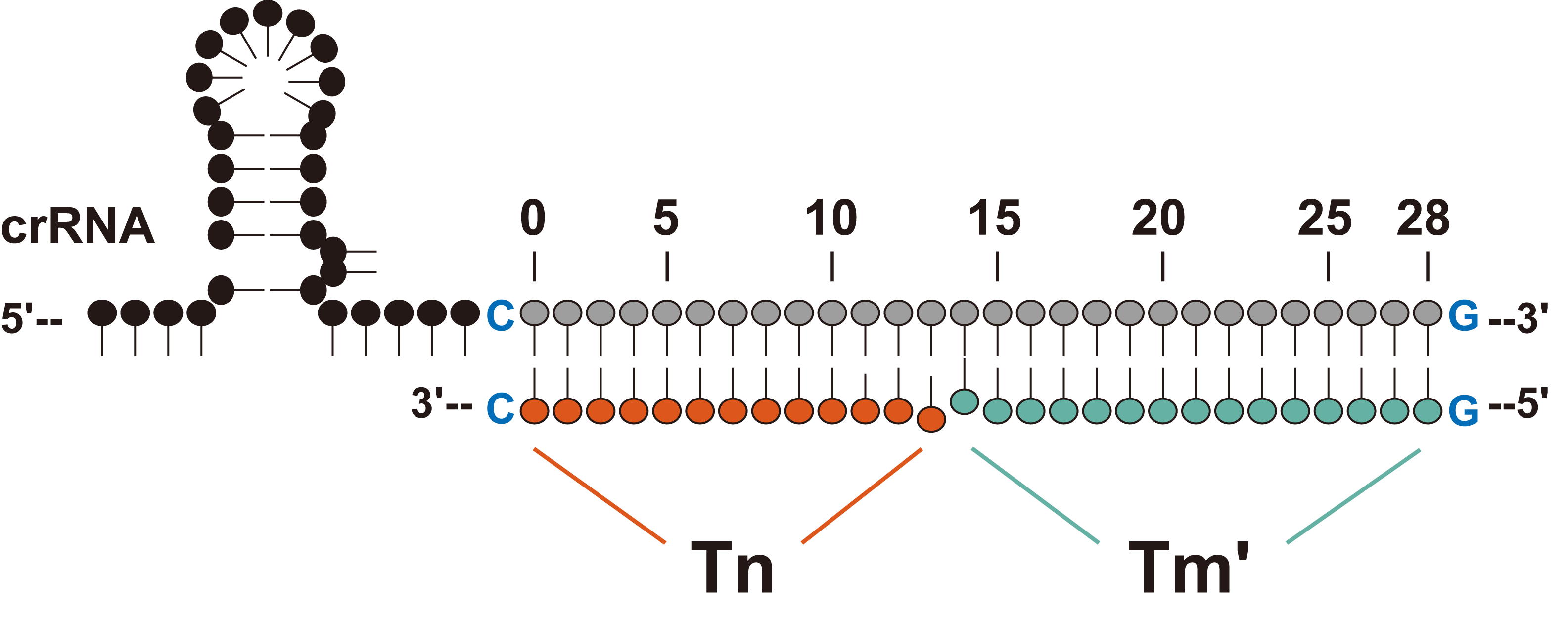
**

Figure S1. Schematic diagram of the dual-effector combination: Tn + Tm'.


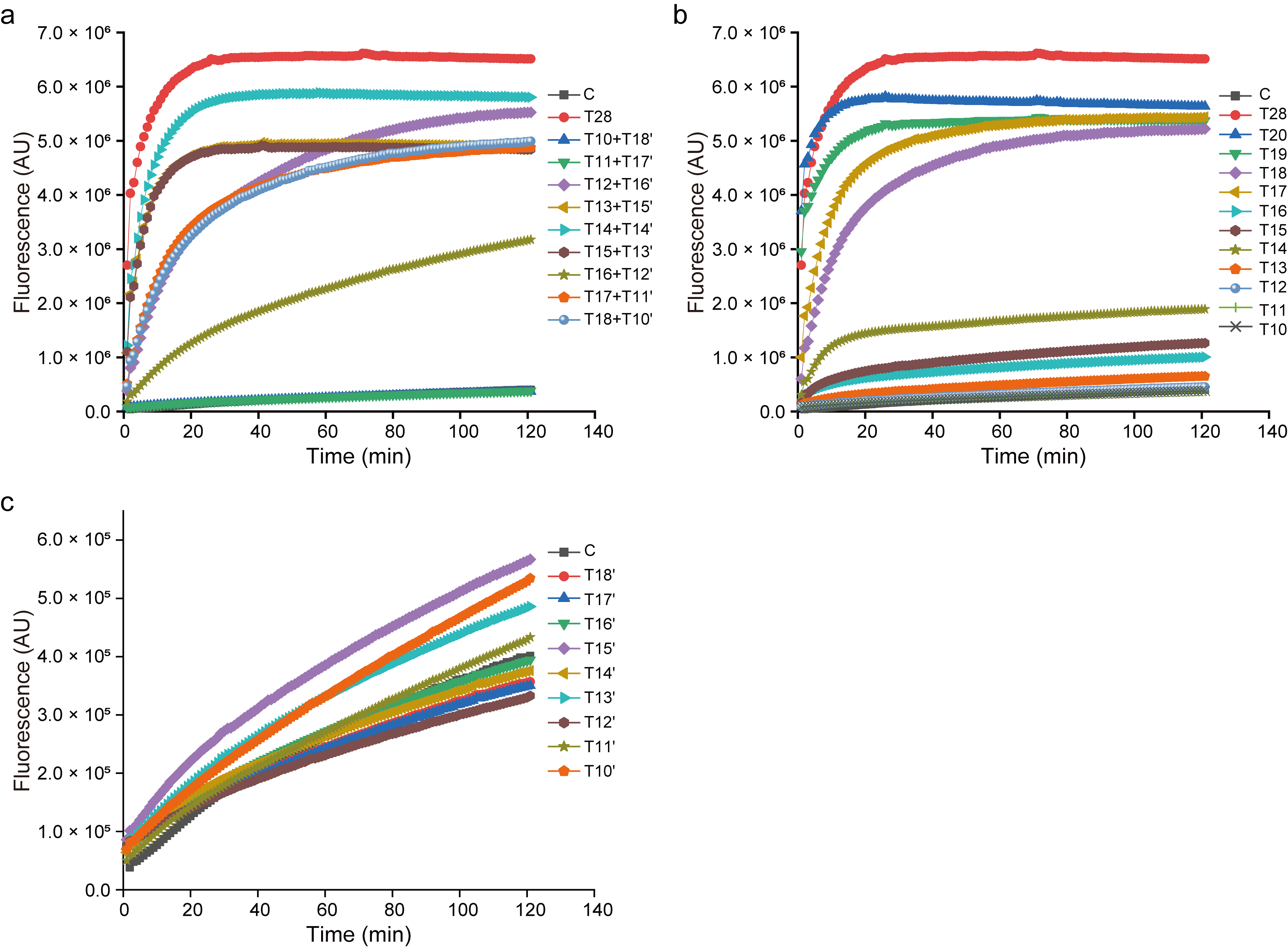


Figure S2. **Representative time course showing the effect of various double-effector combinations on Cas13a trans-cleavage activity.** (a) Signal from the double-effector combination Tn + Tm'. (b) Signal from Tn alone. (c) Signal from Tm' alone.


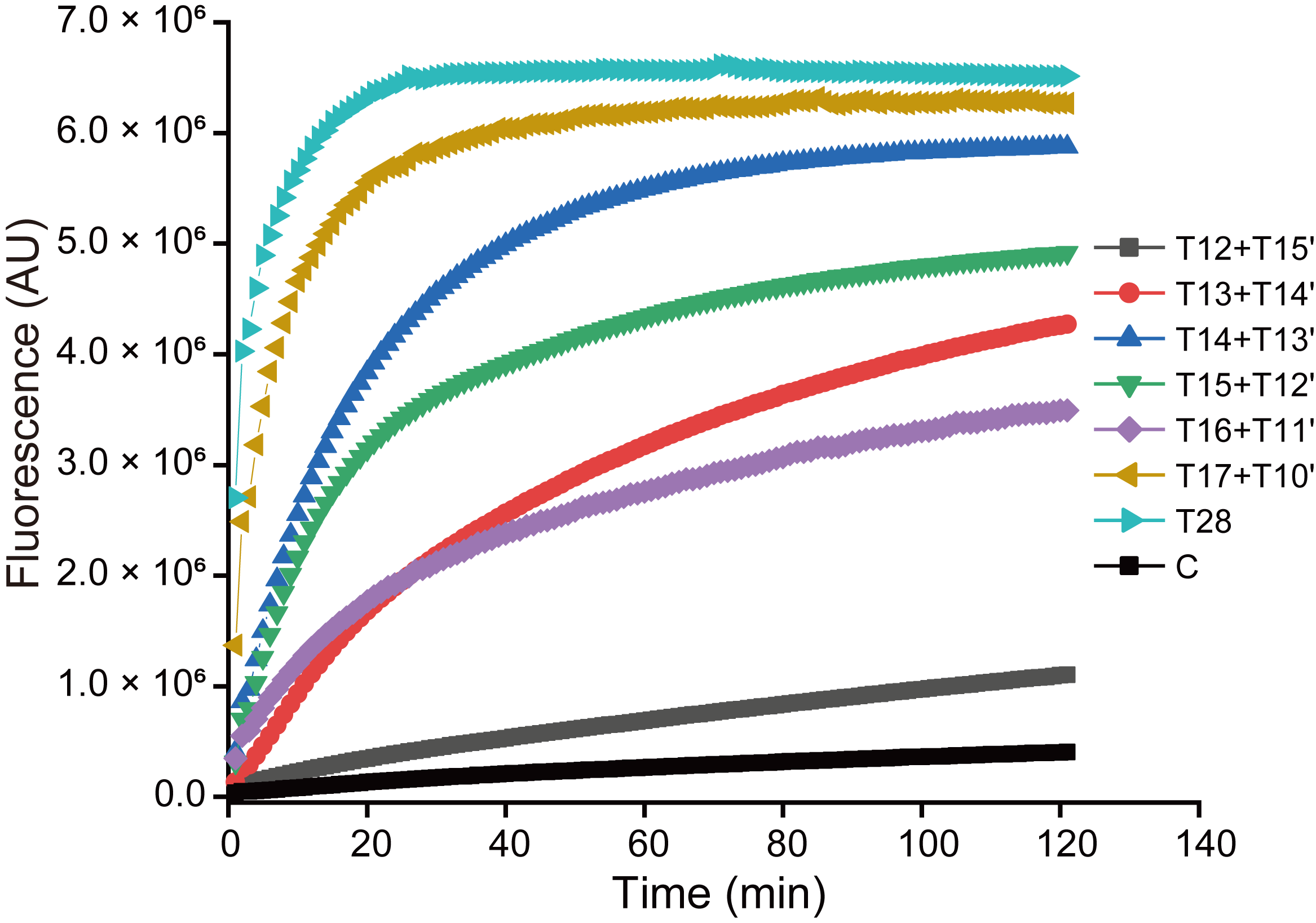


Figure S3. **Representative time course showing the effect of a double-effector combination with a single-base deletion on Cas13a trans-cleavage activity.**


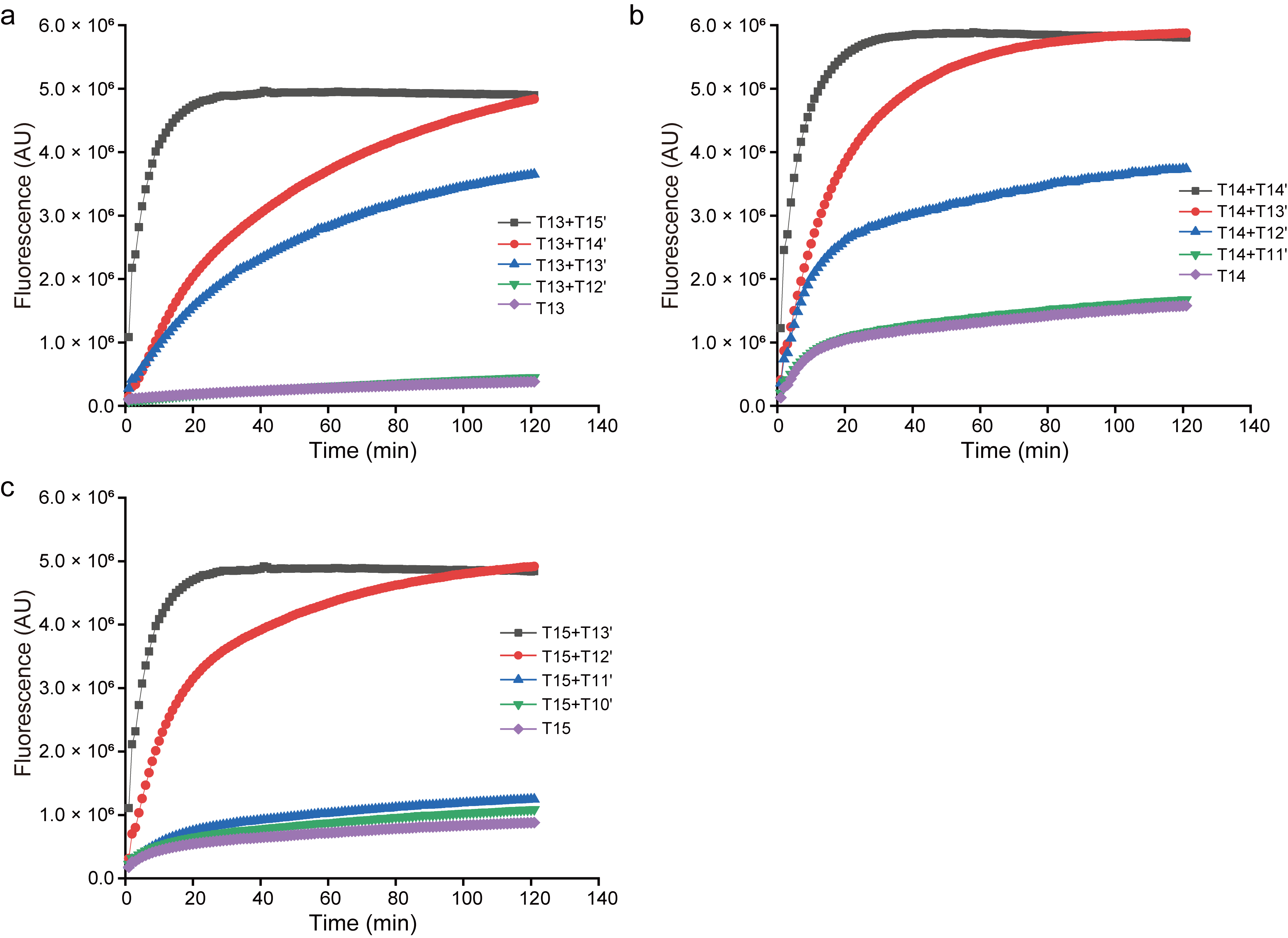


Figure S4. **Representative time course showing the effect of double-effector combinations with different base deletions on Cas13a trans-cleavage activity.** (a) T13 + T15'. (b) T14 + T14'. (c) T15 + T13'.


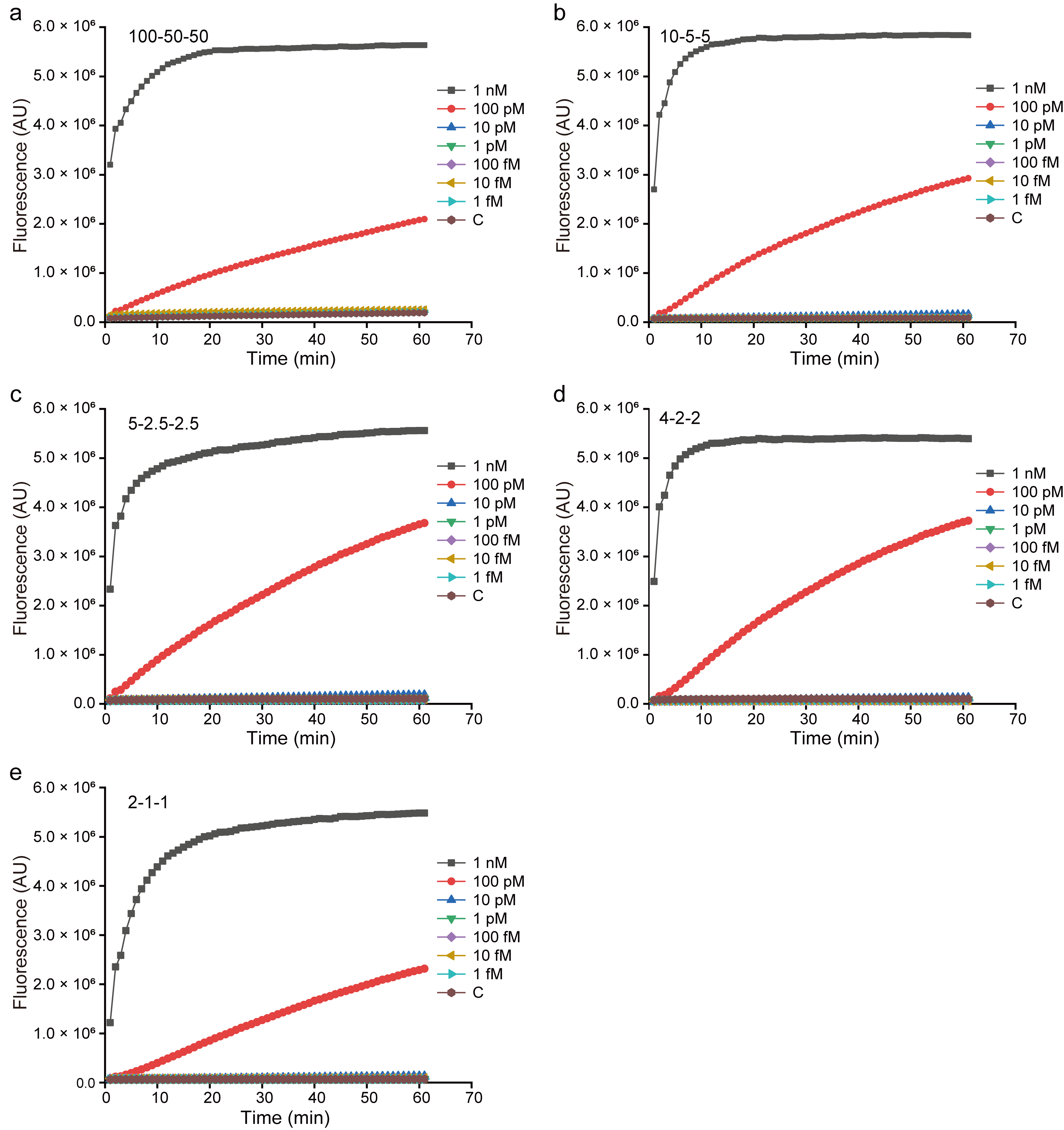


Figure S5. **Representative time course showing the effect of premixed solution concentrations on the sensitivity of Cas13a in detecting unilateral target T13.** (a) The premixed solution contained 100 nM Cas13a, 50 nM crRNA, and 50 nM T15' (denoted as 100-50-50). (b) 10-5-5. (c) 5-2.5-2.5. (d) 4-2-2. (e) 2-1-1.


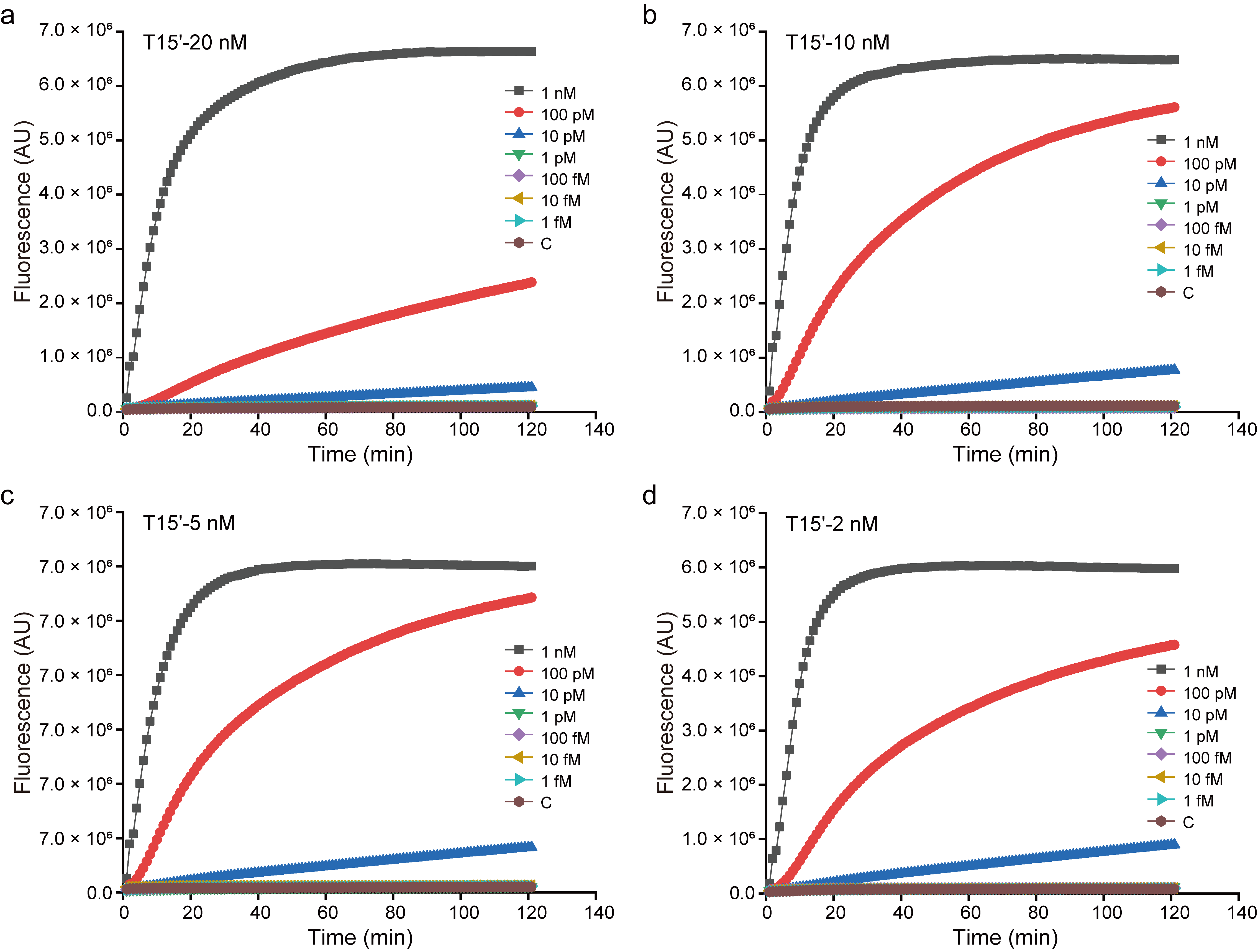


Figure S6. **Representative time course showing the effect of varying T15' concentrations on the sensitivity of Cas13a in detecting the unilateral target T13.** (a) 20 nM. (b) 10 nM. (c) 5 nM. (d) 2 nM.


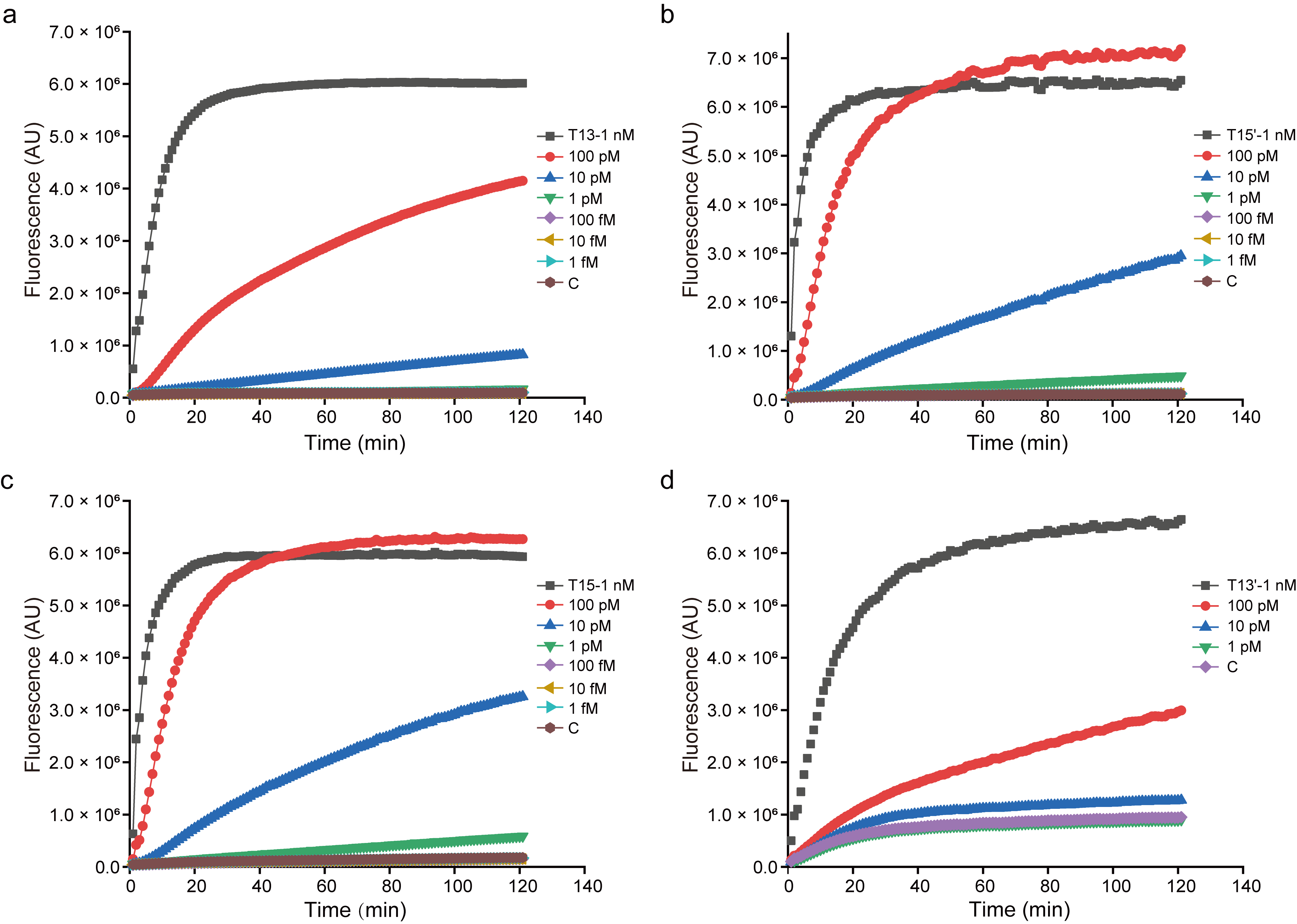


Figure S7. **Representative time courses of CRISPR/Cas13a-CTAM sensitivity for unilateral target detection in T13 + T15' and T15 + T13' double-effector combinations.** (a) Detection of T13 with T15' fixed. (b) Detection of T15' with T13 fixed. (c) Detection of T15 with T13' fixed. (d) Detection of T13' with T15 fixed.


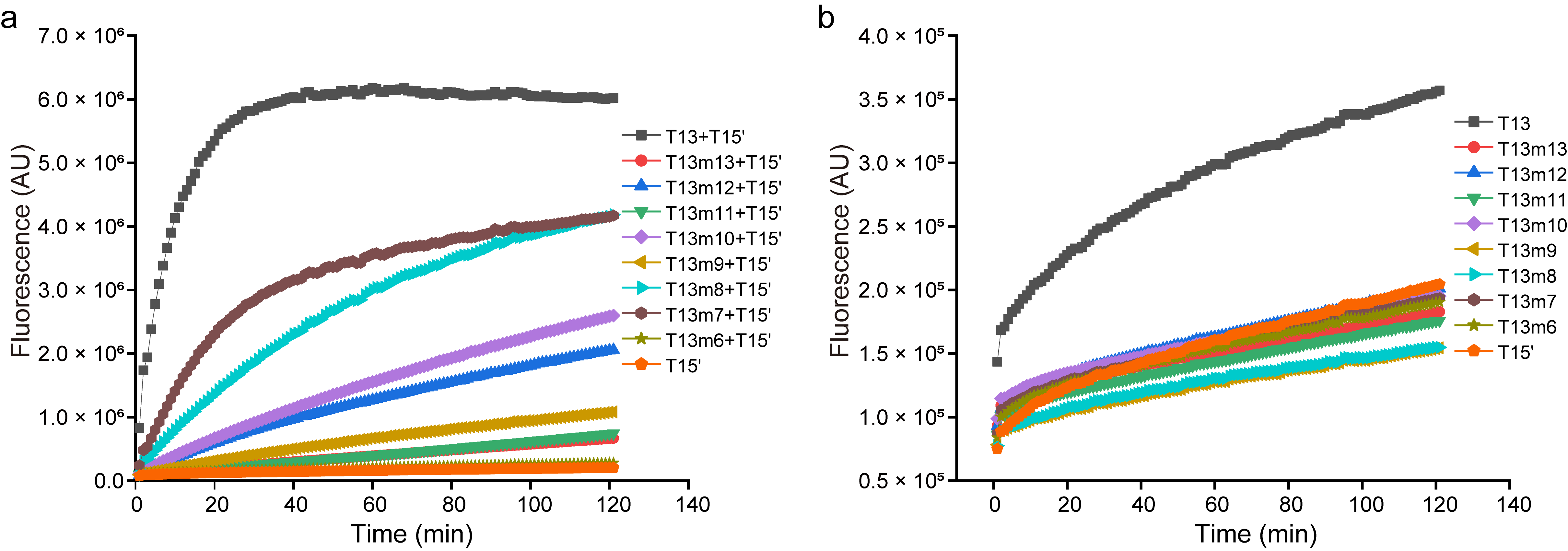


Figure S8. **Representative time course showing the effect of the T13 target with a single-base mismatch (T13mN) on Cas13a trans-cleavage activity.** (a) T13mN cooperates with T15' to activate Cas13a trans-cleavage activity. (b) Background signals of T13mN, all of which are lower than those of T13.


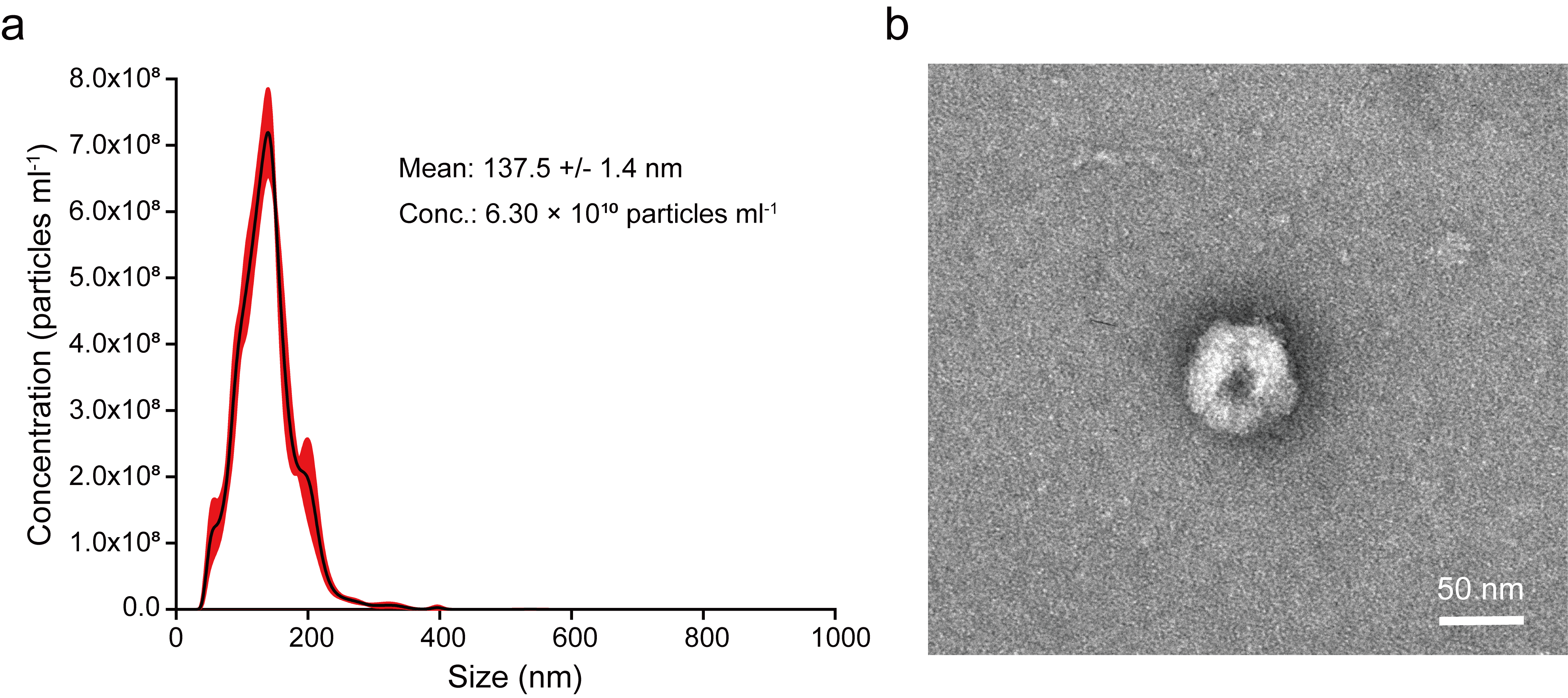


Figure S9. **Characterization of exosomes purified by ultracentrifugation:** (a) NTA analysis; (b) TEM imaging.


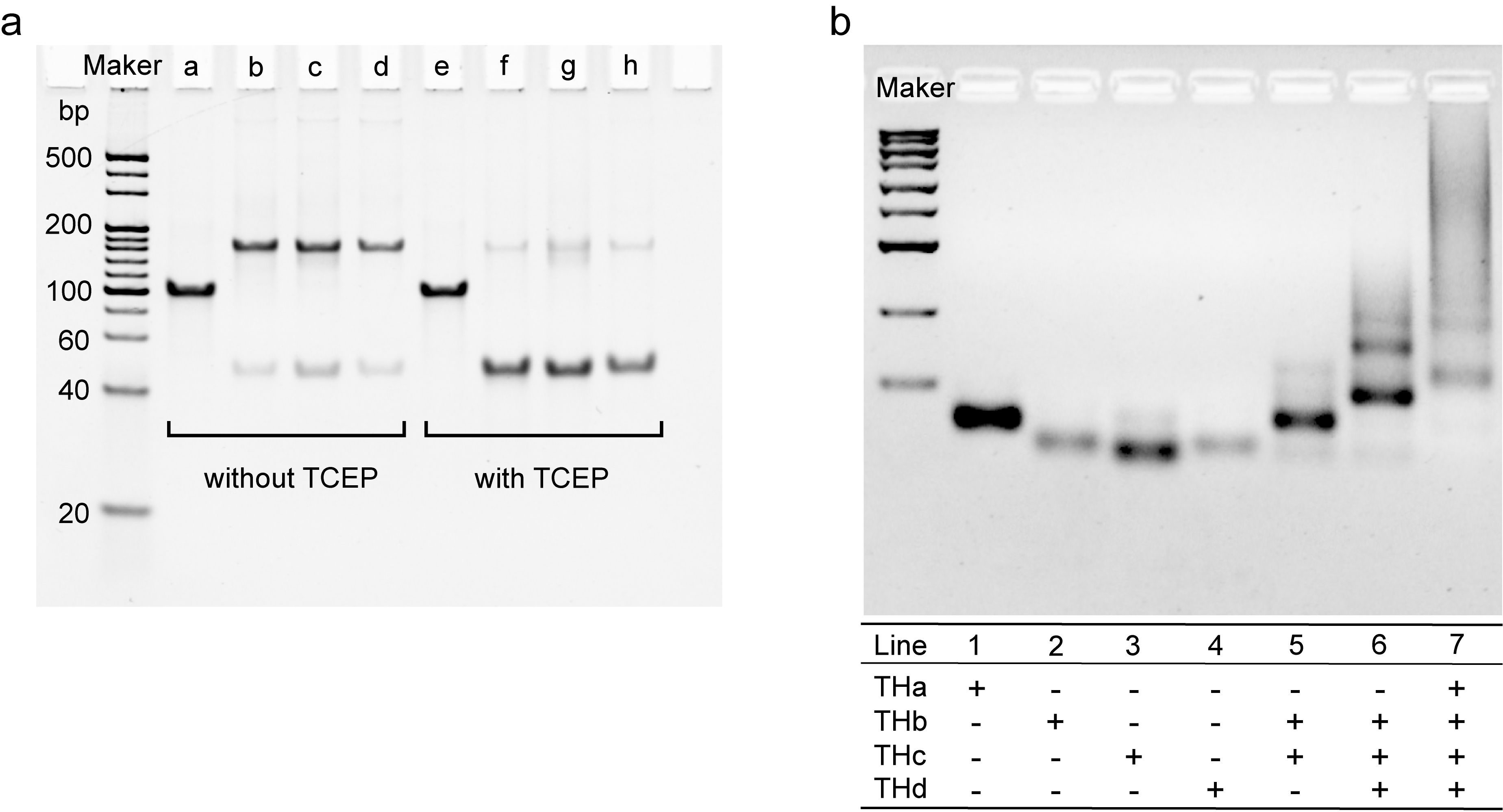


Figure S10**. Pretreatment and assembly of TDNA were characterized using 12.5% PAGE and 2.5% agarose gel electrophoresis, respectively.** (a) Pretreatment of the TDNA thiol monomer with TCEP reduces unwanted disulfide bonds. (b) As the assembly of monomers progresses, the migration rate of TDNA and its intermediates gradually slows.

# SUPPLEMENTARY TABLES

**Table S1.** The sequences used in the theoretical study of CRISPR/Cas13a-CTAM.


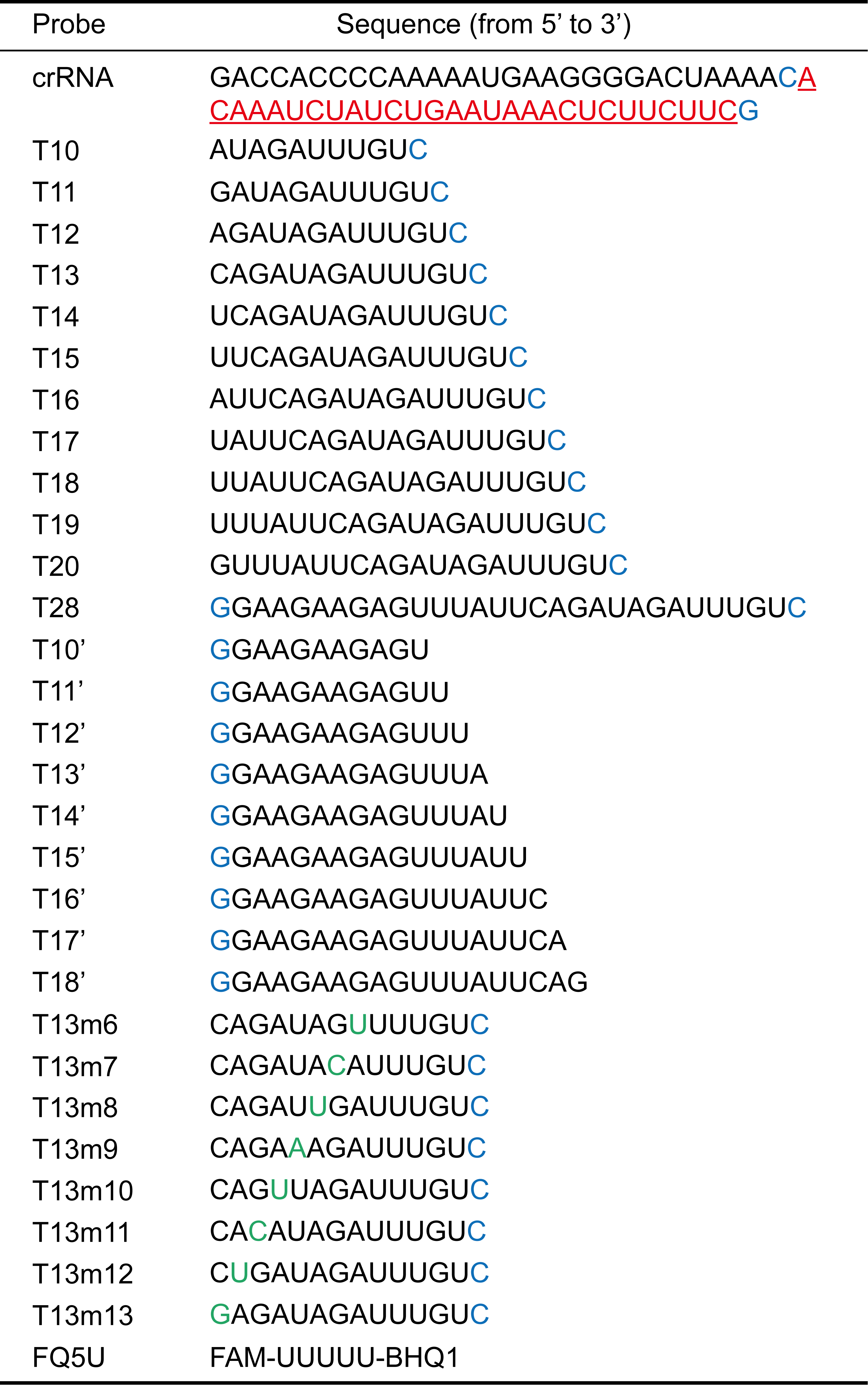


Underline: Spacer sequence; Green: Mismatched bases of T13 target; C and G: The -1th and 29th base.

Table S2. The sequences used in the study of combined detection of miR-155 and miR-499 based on CRISPR/Cas13a-CTAM.


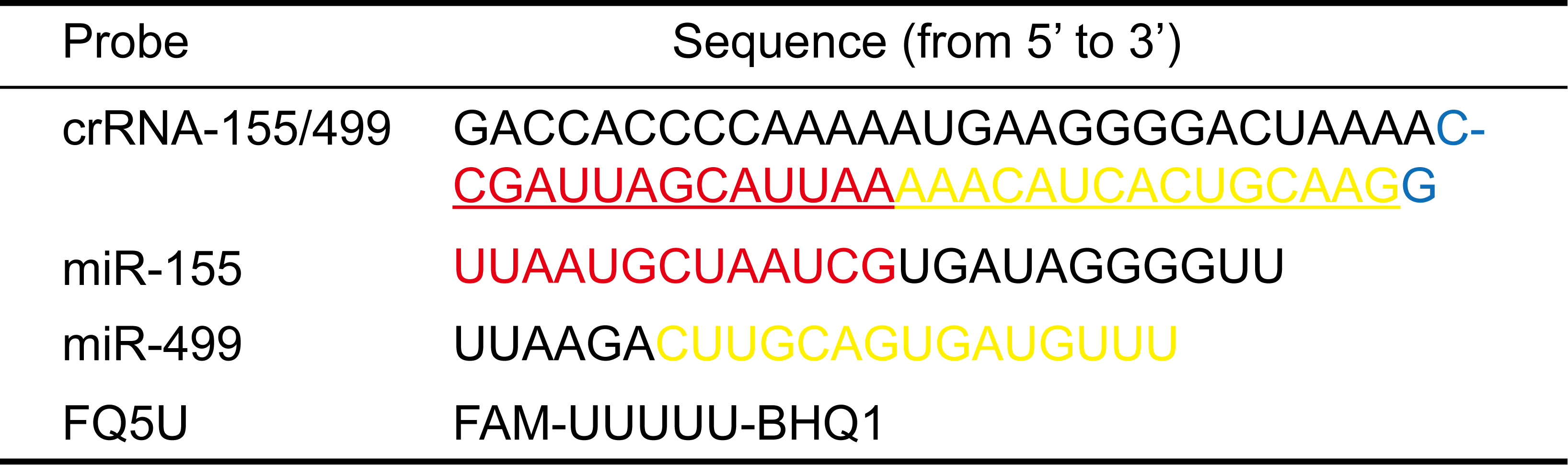


Underline: Spacer sequence of crRNA; Red: Complementary sequence between spacer and miR-155; Yellow: Complementary sequence between spacer and miR-499; C and G: The -1th and 29th base.

Table S3. The sequences used in the study of the combined detection of adjacent proteins on the membrane surface of exosome based on CRISPR/Cas13a-CTAM.

**
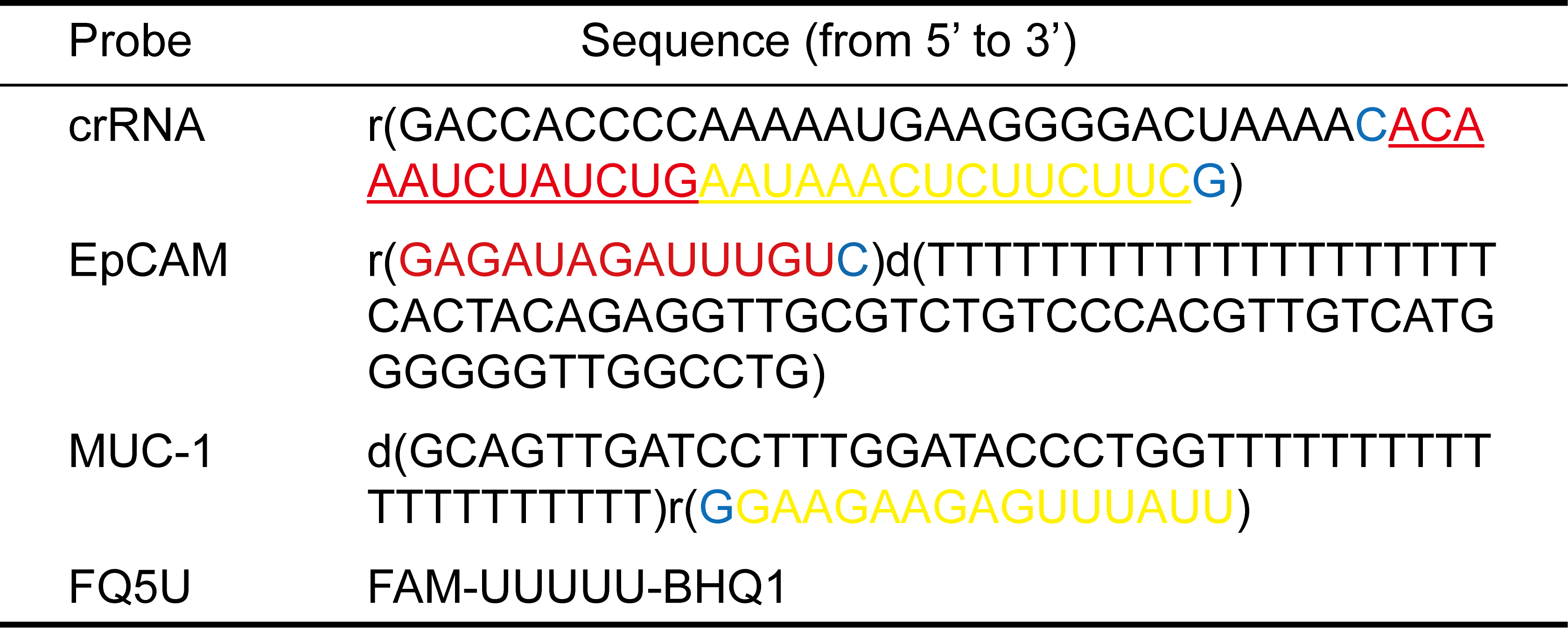
**

Underline: Spacer sequence of crRNA; Red: Complementary sequence between spacer and EpCAM aptamer 5' extension segment; Yellow: Complementary sequence between spacer and MUC-1 aptamer 5' extension segment; C and G: The -1th and 29th base.

Table S4. The sequences used in the study of the in-situ detection of miRNA at the sensor interface based on CRISPR/Cas13a-CTAM immobilized Cas13a.


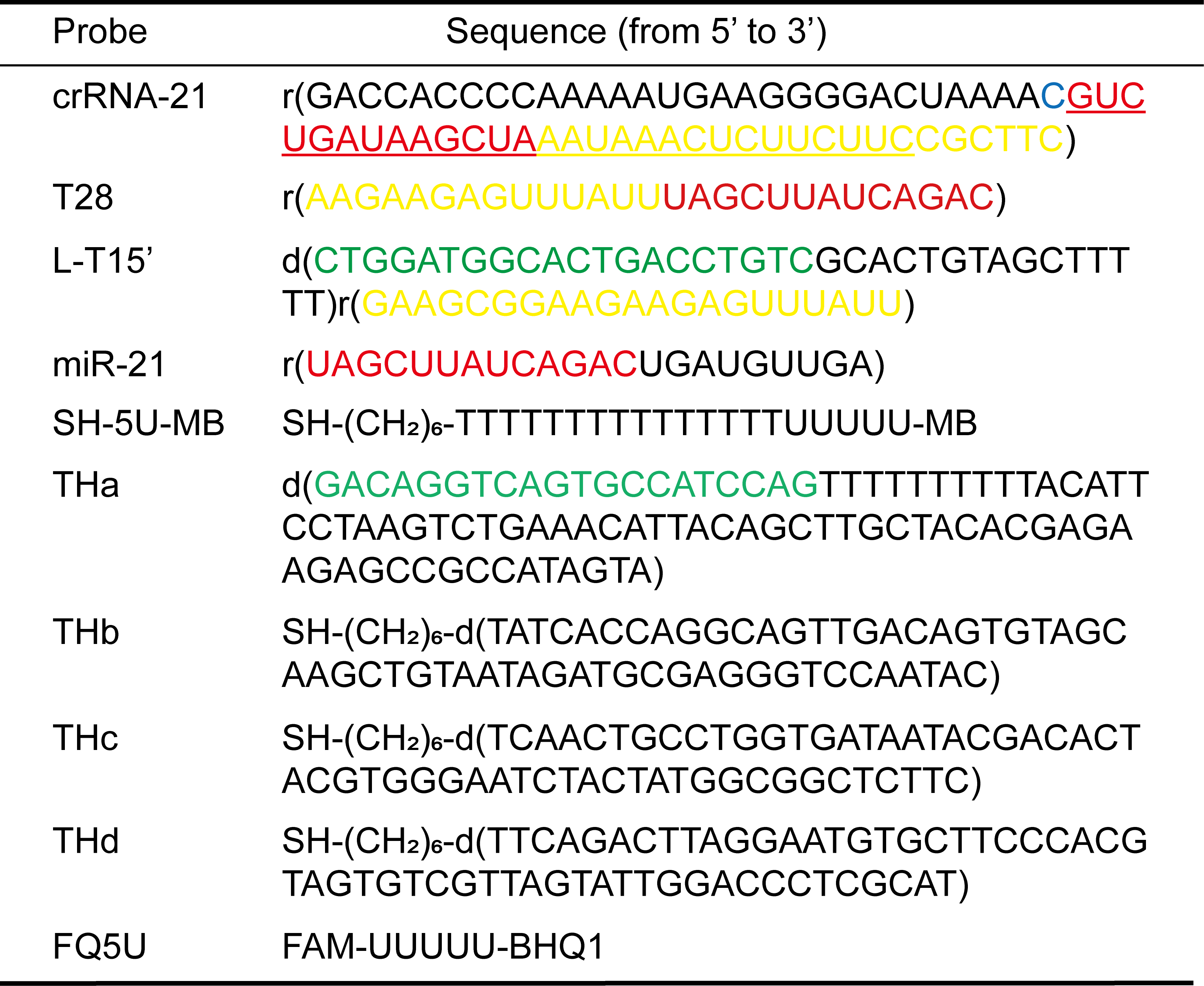


Underline: Spacer sequence of crRNA; Red: Complementary sequence between spacer and miR-21; Yellow: Complementary sequence of spacer sequence and its extended segment with L-T15'; Green: Complementary sequence of L-T15' with THa; C and G: the -1th and 29th base.

Table S5. Electrochemical detection stability and reproducibility.


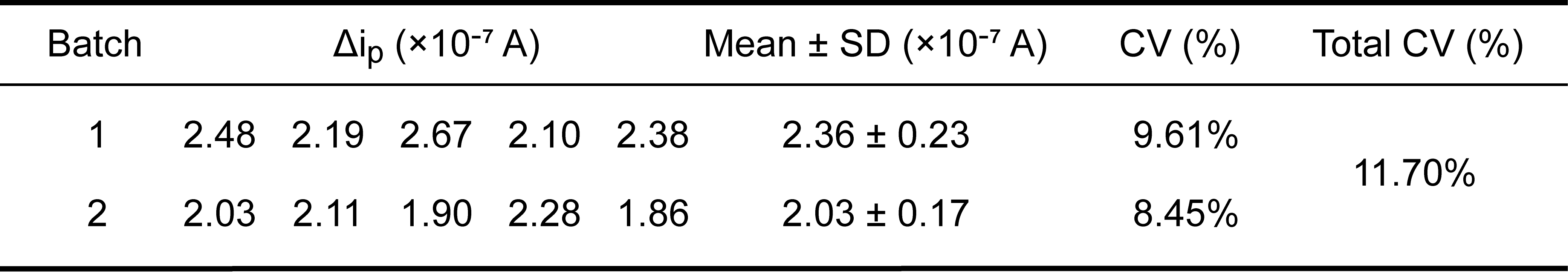

Supplement: Supplementary file 1 — Supporting File: advs74968‐sup‐0001‐SuppMat.docx. [file ADVS-13-e24156-s001.docx]
